# Microscale Neuronal Activity Collectively Drives Chaotic and Inflexible Dynamics at the Macroscale in Seizures

**DOI:** 10.1523/JNEUROSCI.0171-22.2023

**Published:** 2023-04-05

**Authors:** DRW Burrows, G Diana, B Pimpel, F Moeller, MP Richardson, DS Bassett, MP Meyer, RE Rosch

**Affiliations:** 1 MRC Centre for Neurodevelopmental Disorders, King’s College London, London, UK; 2 Department of Neurophysiology, Great Ormond Street Hospital NHS Foundation Trust, London, UK; 3 GOS-UCL Institute of Child Health, University College London, London, UK; 4 Department of Bioengineering, University of Pennsylvania, Philadelphia PA, USA; 5 Department of Electrical & Systems Engineering, Physics & Astronomy, Neurology, and Psychiatry University of Pennsylvania, Philadelphia PA, USA; 6 Santa Fe Institute, Santa Fe NM, USA

## Abstract

Neuronal activity propagates through the network during seizures, engaging brain dynamics at multiple scales. Such propagating events can be described through the avalanches framework, which can relate spatiotemporal activity at the microscale with global network properties. Interestingly, propagating avalanches in healthy networks are indicative of critical dynamics, where the network is organised to a phase transition, which optimises certain computational properties. Some have hypothesised that the pathological brain dynamics of epileptic seizures are an emergent property of microscale neuronal networks collectively driving the brain away from criticality. Demonstrating this would provide a unifying mechanism linking microscale spatiotemporal activity with emergent brain dysfunction during seizures. Here, we investigated the effect of drug-induced seizures on critical avalanche dynamics, using *in vivo* whole-brain 2-photon imaging of GCaMP6s larval zebrafish (males and females) at single neuron resolution. We demonstrate that single neuron activity across the whole brain exhibits a loss of critical statistics during seizures, suggesting that microscale activity collectively drives macroscale dynamics away from criticality. We also construct spiking network models at the scale of the larval zebrafish brain, to demonstrate that only densely connected networks can drive brain-wide seizure dynamics away from criticality. Importantly, such dense networks also disrupt the optimal computational capacities of critical networks, leading to chaotic dynamics, impaired network response properties and sticky states, thus helping to explain functional impairments during seizures. This study bridges the gap between microscale neuronal activity and emergent macroscale dynamics and cognitive dysfunction during seizures.

## Introduction

Epilepsy is a collection of neurological syndromes primarily defined by the recurrence of seizures (Fisher et al., 2014). Seizures can acutely impair awareness (Blumenfeld, 2012), and patients with uncontrolled seizures (~1/3) (Kwan & Brodie, 2000) experience severely reduced quality of life (Sperling, 2004). Seizures are characterised by abnormal brain dynamics at multiple scales, engaging neurons and glia (Diaz Verdugo et al., 2019; Khoshkhoo et al., 2017; Magloire et al., 2019), neuronal ensembles (Liu & Baraban, 2019; Muldoon et al., 2013), and coarse brain areas (Gibbs et al., 1935), with non-trivial relationships between micro and macroscale dynamics (Martinet et al., 2017).

One approach to linking microscale collective behaviours during seizures with emergent properties at larger scales is statistical physics, which estimates microscopic variables probabilistically to study macroscopic properties. In particular, quantifying how systems respond to perturbations with spreading bursts of activity, known as avalanches (Bak et al., 1987), can be informative about the global organisation of the network (Sethna et al., 2001). For example, avalanche dynamics in the brain indicate that healthy neuronal networks are organised at a phase transition, called criticality, which is computationally favourable (Beggs & Plenz, 2003; Ponce-Alvarez et al., 2018). The avalanches framework is also relevant to seizure dynamics, which consist of slowly spreading wavefronts of bursting activity (Schevon et al., 2012; Trevelyan et al., 2006), from which fast, depolarising potentials propagate outwards (Martinet et al., 2017; Smith et al., 2016). Interestingly, macroscopic seizure recordings suggest that coarse propagating events give rise to exponentially growing avalanches (Arviv et al., 2016; Harris, 1963; Meisel et al., 2012), fuelling speculation that neuronal activity drives the network away from criticality. Although hyper-synchronous burst firing in the ictal core resembles such avalanche expansion (Weiss et al., 2013), evidence for heterogeneous spatial recruitment (Lau et al., 2022; Muldoon et al., 2013) and asynchronous neuronal activity during seizures (Aeed et al., 2020; Truccolo et al., 2011), suggest a more complicated picture at the microscale. Therefore, it remains uncertain whether microscale avalanche behaviour collectively drives network dynamics away from a phase transition during epileptic seizures.

Importantly, the proximity of networks to criticality can be used to infer emergent computational properties relevant to brain pathology (Hesse & Gross, 2014). In particular, systems at criticality can encode a diversity of inputs (Kinouchi & Copelli, 2006), flexibly explore many brain states, and reside between sub-optimal regimes of chaos and order (Haldeman & Beggs, 2005). Therefore, a departure from criticality could explain emergent network dysfunction that occurs during seizures. For example, such a ‘supercritical’ regime, could explain the stereotyped population dynamics of the seizureonset zone (Liu et al., 2018), the reduced entropy of brain states (Ren et al., 2021) and the chaotic behaviour (Babloyantz & Destexhe, 1986) that arises during seizures. In this way, a loss of criticality may provide a unifying mechanism through which microscale propagating activity drives emergent pathological dynamics during seizures.

Here, we studied avalanche dynamics during seizures to investigate criticality and emergent computational properties in seizure networks. Importantly, micro and macroscale seizure recordings often show disparate dynamics, due to low spatial resolution of macroscale recordings (Meyer et al., 2018; Wenzel et al., 2019), and the impact of subsampling neuronal activity at the microscale (Priesemann et al., 2009). To address these concerns, we take advantage of the transparency of larval zebrafish to perform *in vivo* functional imaging of the whole brain at single-cell resolution during seizures (Ahrens et al., 2013; Burrows et al., 2020; Hadjiabadi et al., 2021). We show that brain-wide, single neuron activity collectively drives macroscale dynamics away from criticality. We then use spiking network models to demonstrate that only densely connected networks can drive the network away from criticality during seizures. Importantly, such dense networks also disrupt the optimal computational capacities of critical networks, helping to explain functional impairments during seizures. This study bridges the gap between microscale neuronal activity and emergent macroscale dynamics and brain dysfunction during seizures.

## Materials and Methods

### Experimental Models

In order to capture brain-wide seizure activity at cellular resolution, we took advantage of the optical transparency of the larval zebrafish. Transgenic zebrafish larvae, *Tg(elavl3:H2B-GCaMP6s)*, expressing a nuclear-localised, genetically-encoded calcium sensor pan-neuronally, were used to capture neuronal dynamics (gift from Misha Ahrens, Janelia Research Campus) (J. Freeman et al., 2014). To maximise optical transparency, *Tg(elavl3:H2B-GCaMP6s)* larvae were crossed with melanophore deficient (-/-) roy;nacre mitfa mutants (Lister et al., 1999). Zebrafish larvae were raised at 28°C in danieau solution on a day and night cycle of 12:12 hours. All imaging experiments were performed at 6 days post fertilisation (dpf), using fish of either sex. This work was approved by the local Animal Care and Use Committee (Kings College London) and was carried out in accordance with the Animals (Experimental Procedures) Act, 1986, under license from the United Kingdom Home Office.

### 2-photon Calcium Imaging

**Figure 1:**
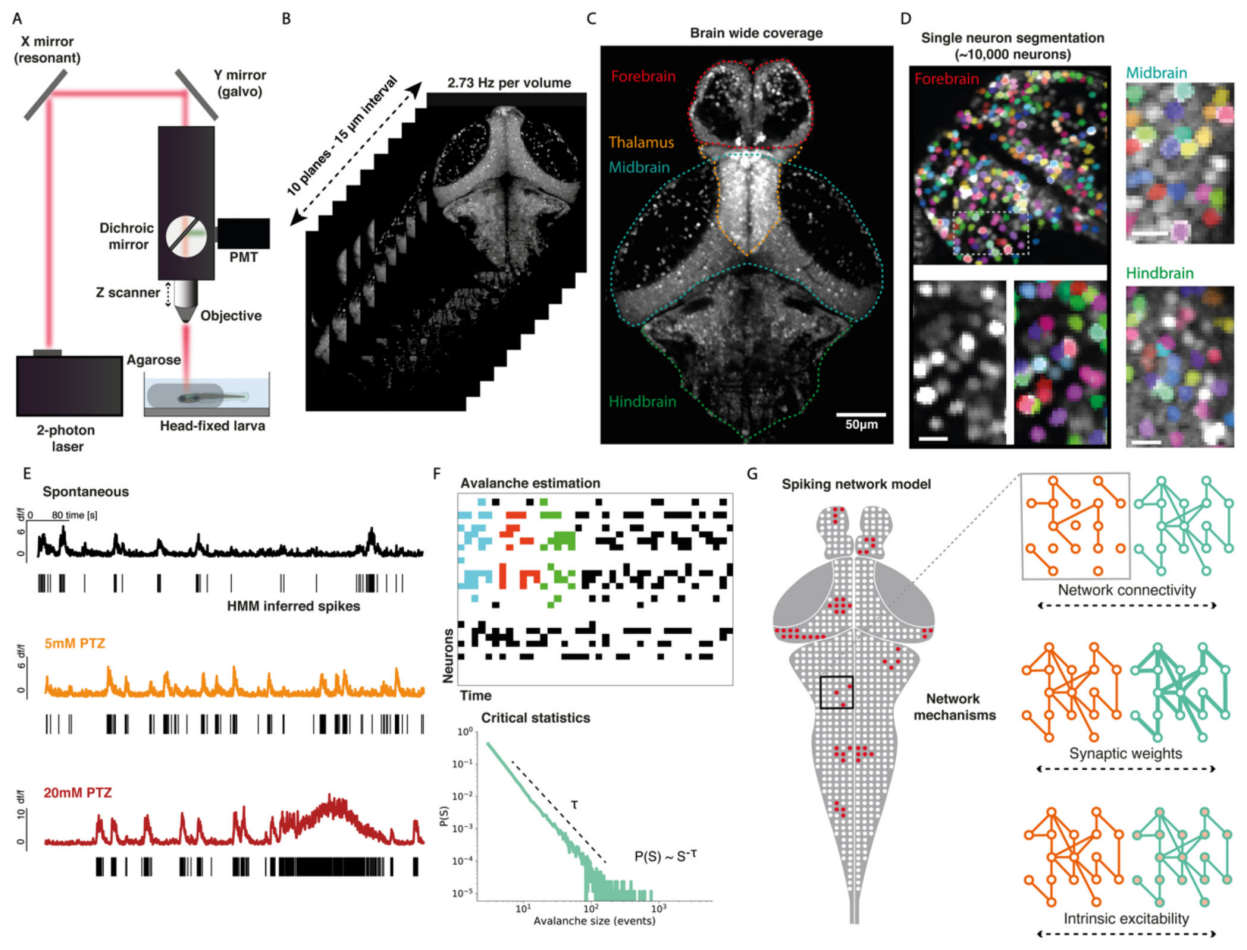
Study design. (A) In vivo 2-photon imaging setup with head-fixed larval zebrafish. (B) Imaging was captured across 10 planes with 15 μm spacing at an imaging rate of 2.73 Hz per volume. (C) Max projection across 2 imaging planes of larval zebrafish volume taken with 2-photon microscope, demonstrating coverage of major brain regions. (D) Nuclear localised GCaMP fluorescence enables the segmentation of single neurons, as shown over exemplar forebrain (left), midbrain (top right) and hindbrain areas (bottom right) for a representative fish. The two images below the forebrain region show raw GCaMP signal (left) and segmented neurons (right) over a magnified area (scale bar is 10 μm). (E) Single cell traces shown from representative neurons for a single fish, showing normalised calcium fluorescence over time for *spontaneous* (black), *5mM PTZ* (orange) and *20mM PTZ* (red) conditions. A hidden Markov model (HMM) was used to infer spike times (black bars). (F) The spatio-temporal propagation of activity through the network was quantified as avalanches, as shown for 3 example avalanches (coloured by avalanche) for an example raster plot (top). Avalanche statistics were calculated to assess critical dynamics (bottom). (G) A network model of the larval zebrafish brain was constructed, which was used to test the role of specific network mechanisms in driving empirical avalanche dynamics.

In order to record brain activity we performed 2-photon calcium imaging of *Tg(elavl3:H2B-GCaMP6s)*, (-/-) roy;nacre larval zebrafish. Non-anaesthetised larvae at 6 dpf were immobilised in 2% low-melting point agarose (Sigma-Aldrich) and mounted dorsal side up on a raised glass platform that was placed in a custom-made Danieau-filled chamber. All imaging was performed on a custom built 2-photon microscope (Independent NeuroScience Services, INSS), which utilises a Mai Tai HP ultrafast Ti:Sapphire laser (Spectraphysics) tuned to 940nm. Objective laser power was at 15mW for all experiments. Emitted light was collected by a 16x, 0.8NA water immersion objective (Nikon) and detected via a gallium arsenide phosphide detector (ThorLabs). Scanning was performed by a resonance scanner (x-axis) and galvo-mirror (y-axis), with a piezo lens holder (Physik Intrumente) adjusting the z-plane (Figure 1A). Images were acquired at a resolution of 512x512 pixels, with a pixel size of 1.05 × 1.03 μm. Single planes were imaged at 30 Hz, with a pixel dwell time of 88ns. Volumetric data was collected across 10 planes at 15μm intervals, with a flyback time of 17ms and actuator lag of 10ms, resulting in a frame rate of 2.73 Hz per volume with 4,914 volumes collected per imaging block (Figure 1B). 15μm intervals were chosen to minimise the possibility for double-identification of neurons across planes – studies investigating cell body size in larval zebrafish suggest cell diameters smaller than 15μm (J. C. Liao & Haehnel, 2012).

### Seizure Induction

To image seizure dynamics with the 2-photon, we administered the GABA_A_ antagonist pentylenetetrazole (PTZ) to larval zebrafish. PTZ elicits clonus-like convulsions, epileptiform discharges (Baraban et al., 2005), which are removed with conventional anti-seizure medication (Afrikanova et al., 2013; Berghmans et al., 2007). To capture baseline and seizure dynamics we recorded 3 × 30 minute consecutive imaging blocks for each fish: 1) *spontaneous* activity representing normal dynamics (Movie 1), 2) *5mM PTZ* causing focal hyper-excitability (Movie 2), and 3) *20mM PTZ* causing highly synchronous, brain-wide hyper-excitability suggestive of generalised seizures (Movie 3) (Figure 1D). Here we refer to generalised network activity as generalised seizures, but it is important to note that these dynamics are not necessarily clinically-defined generalised seizures. PTZ was added into the imaging chamber immediately after each session via a 1ml dose of PTZ suspended in danieau, after which point imaging was restarted. Data was collected and analysed in 10 fish.

### 2-photon Image Processing

In order to derive single neuron activity from 2-photon images, we performed quality control, registration and segmentation steps. Registration and segmentation processing steps were performed across each plane separately. Firstly, recordings were corrected for drift in x and y planes by using the rigid registration algorithm on suite2p (Pachitariu et al., 2017). Next, we applied the segmentation algorithm on suite2p, which identifies contiguous, correlated pixels enabling the identification of nuclear-localised GCaMP signals (Figure 1D). We selected the diameter of nuclei for segmentation as 5 pixels, corresponding to ~5 μm. 5 pixels was chosen as it best matched the size of single nuclei in our observed imaging data. Importantly, 5μm lies within the reported diameter of neuronal nuclei as reported in previous studies at similar ages in larval zebrafish (~3-10μm) (Bruzzone et al., 2021; Kim et al., 2014; Migault et al., 2018).

We found that the segmentation process identified some false positive cells due to noise. To remove these non-cells, a false-positive detection algorithm was designed which relies on the assumption that true cells would show fluorescence spikes with a slower decay time than shot noise events, due to the decay time of GCaMP6s. The false-positive detection algorithm works as follows: 1) split the trace for each cell into 9 frame windows, 2) find the minimum fluorescence value across each window, 3) find the single maximum value across all minimum window values for each cell. This process creates a distribution of max-of-min fluorescence window values (a measure of the fluorescence decay speed), for all segmented cells across the brain. A threshold was then chosen to remove non-cells for each fish. This process enabled the accurate segmentation of ~10,000 neurons per fish.

### Calcium Transient Estimation

In order to estimate calcium events we applied a hidden Markov model to single neuron fluorescence traces, using a method previously developed by our group (Diana et al., 2019). This method enables the robust estimation of the onset of calcium transients, and labels each timepoint for a given neuron as either on or off. The model requires the selection of the parameter *q*, defining the probability of an event occurring. This parameter’s value was chosen by visual inspection of which *q* gave the most accurate representation of calcium traces in our data (*q* = 0.59) (Figure 1E).

### Identification of Seizure State Transitions

To examine neuronal dynamics just before and right after seizure onset, we identified timepoints where seizure transitions occurred. To clearly delineate pre and post seizure periods, we took advantage of the characteristic brain dynamics during *20mM PTZ* administration, where the brain abruptly transitions into a generalised seizure state (Diaz Verdugo et al., 2019). To define the transition timepoint for each fish, we moved a 30 frame sliding window over the mean activity across all neurons. By finding the window with the maximum difference between the starting timepoint and the subsequent 29 frames, we could identify the time window in which the greatest activity increase occurs (i.e. the generalised seizure transition). To compare dynamics across state transitions, we compared activity from 400 frames just before the generalised seizure (*pre-ictal*), the first 400 frames of the generalised seizure (*ictal-onset*) and 400 frames from a randomly selected segment of spontaneous recording (*baseline*).

### Neuronal Avalanche Estimation

We were interested in describing the population dynamics of neuronal activity at the microscale. A key form of collective behaviour in certain systems, is the propagation of activity through the network, often described as avalanches (Bak et al., 1987) (Figure 1F). Evidence from brain recordings suggest the presence of avalanches in brain activity, termed neuronal avalanches (Beggs & Plenz, 2003). Here, we apply the avalanches framework to study the propagation of brain activity under both physiological and pathological (seizure) conditions. In order to capture avalanches we estimated the spatio-temporal propagation of activity through the network, using the binarised activity traces across all neurons. Given that the underlying synaptic connectivity in our network is unknown, we infer the flow of activity from an *n* by *m* matrix, where *n* represents each neuron and *m* is the on/off state at timepoint *m*. To do this we adapt methods used in previous studies to estimate avalanche size and duration (Ponce-Alvarez et al., 2018; Tagliazucchi et al., 2012).

**Figure 2:**
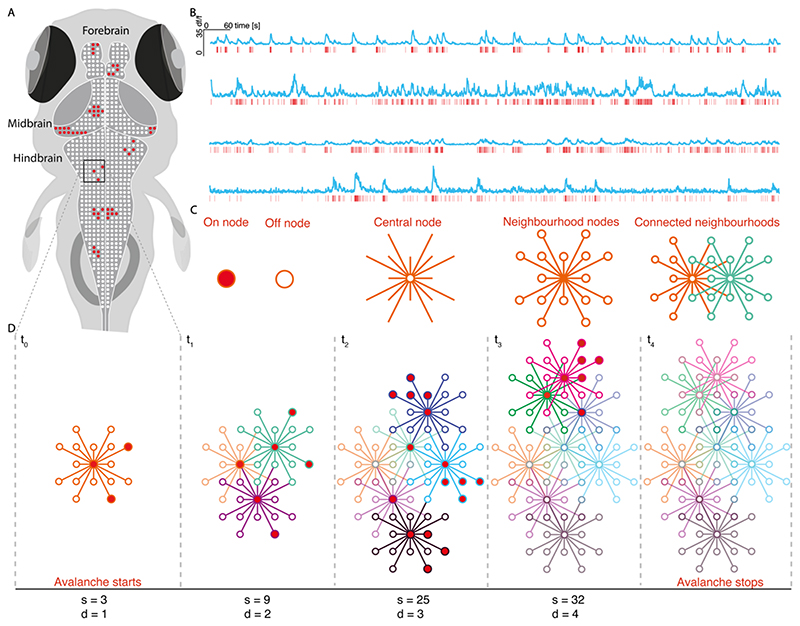
Avalanche estimation. (A) Neurons exhibit on (red circles) and off (white circles) dynamics giving rise to ensembles that grow in space and time. (B) Hidden Markov model estimation of calcium transients showing normalised traces (blue), and estimated calcium transients (red). (C) Avalanche calculation legend. (D) For avalanches to begin, at least 3 nodes within the same neighbourhood must be active at *t_0_*. To propagate in time, any avalanche node active at *t_0_* must also be active at *t_1_*. Once this step is satisfied, any nodes active at *t_1_* that are connected via a neighbourhood to avalanche nodes at *t_1_* become avalanche members. Avalanches terminate when no more nodes are active. Avalanche size (s) is the total number of activations and duration (d) is the number of time steps of the avalanche.

First, we assume that neuron *u* can only activate other neurons in the population *P* that lie within a neighbourhood *N_u,v_* where *v* is the set of the closest *k*% of cells to *u*. This introduces a spatial constraint to avalanche propagation such that activity can only flow between putatively connected neurons, preventing disparate cascades combining into one large avalanche (Figure 2). An avalanche begins when at least *n* cells within a neighbourhood *N_u,v_* are active at *t_x_*, that were not part of an avalanche in the previous timestep. Here we label the neurons currently active at *t_x_* which belong to the avalanche as the set *A_x_* = {*a,b,c…z*}. All active neurons of *P* at *t_x_* connected to any of *A_x_* via a neighbourhood are included into the set *A_x_*.

At each subsequent step avalanche propagation iterates as follows (Figure 2):

1) If at least one neuron that was part of avalanche *A_x_* is also active at *t_x+1_* then the avalanche continues in time, forming the set *A_x+1_* = {*a,b,c...z*}.
2) Any neurons from *P* active at *t_x+1_* that are connected to any of *A_x+1_* via a neighbourhood are included into the set *A_x+1_*.

Once step 1 is no longer satisfied the avalanche terminates. Avalanches whose active neighbourhoods converge are grouped into a single avalanche. Avalanche size was calculated as the total number of calcium events during the avalanche. Avalanche duration was the number of time steps for which the avalanche was active. Our avalanche approach requires the selection of parameter *k*, defining the percentage of neighbours a neuron can connect to. Although the precise ratio of short to long range synaptic connections in the larval zebrafish is unknown, the few studies of structural connectivity in entire nervous systems have shown a strong preference for local connections (Ahn et al., 2006; B. L. Chen et al., 2006; Cherniak, 1994). Importantly, local connectivity preferences can give rise to the strong local correlations (Betzel, 2020), and the shared tuning properties of local neuronal ensembles (Romano et al., 2015), that are found in the larval zebrafish around 5-8 dpf. While this does not preclude the known presence of long range connections in the brain (Kunst et al., 2019), here we chose to focus on local connections to provide the simplest model of propagation which could reasonably describe observed network dynamics whilst relying on the most probable neuron-to-neuron connections. However, to demonstrate the robustness of our avalanche dynamics to the presence of short versus long-range connections, we studied the effect of varying *k* on spontaneous avalanche dynamics. While changing *k* (0.04% – 3%; ~10um – 50um) altered avalanche dynamics (Figure 4B,C), key markers of criticality (power laws and relationships between avalanche size and duration exponents) were partially conserved across *k* – power laws were present across all *k* values (Figure 4A), while critical exponent relations were maintained for neighbourhoods < 30um (Figure 4D). This suggests that key critical statistics are partially robust to the specific parameterisation of *k* in our avalanche algorithm. That critical exponent relations are lost for large neighbourhoods indicates that network with dense, local and medium-range connections fail to give rise to critical avalanche dynamics, as reported in previous studies (Ponce-Alvarez et al., 2018). For further analyses, *k* was selected (0.16%) so that the mean neighbourhood radius was predominantly local (20.4um ± 0.98), and lay within ranges used in previous studies (<30um). Our avalanche approach also requires the selection of parameter *n*, which defines the minimum number of active neurons required to initiate an avalanche. We explored the effect of changing *n* on avalanche statistics in spontaneous activity (n=2,3,4). While changing *n* did slightly alter avalanche dynamics (Figure 4F,G), avalanche power law relationships and critical exponent relationships were conserved across all parameter values (Figure 4E,H). This indicates that critical statistics in spontaneous activity are not biased by the specific parameterisation of *n* in our avalanche algorithm. For all remaining analyses we set the minimum avalanche initiation size to 3, as used in previous studies.

We note that due to the relatively slow decay time of GCaMP6s (550 ms decay half life), our imaging rate (2.73Hz) is sufficient to capture action potential-induced calcium transients in sampled neurons (T.-W. Chen et al., 2013). However, our imaging rate is too slow to resolve the exact number of spikes giving rise to a calcium transient in each timestep. Instead, neuronal activity can be classified into on/off periods. Furthermore, the typical synaptic delay times (~1ms) are too fast for our imaging setup to resolve the exact sequence of spiking events in the network (Barnes et al., 2015). Nonetheless, due to the slow GCaMP6s decay times all events within a sequence should be detected, but grouped into ~360ms bins. Therefore, our avalanche approach captures the propagation of activity through the network in a temporally downsampled manner, based on the presence or absence of calcium transients at a given time step.

### Avalanche Power Law Estimation

We were interested in using the avalanches framework to infer the proximity of the network to a phase transition, known as criticality. Specifically, criticality occurs when a system is poised at a second order phase transition, between distinct macroscopic regimes of order and disorder (Cocchi et al., 2017). To test for the presence of criticality in avalanche dynamics, we took advantage of the fact that avalanches at criticality are scale-invariant. This means that only at criticality can avalanches span all scales, from small to large, which gives rise to power law distributions of avalanche size and duration (Hesse & Gross, 2014). Specifically, critical systems should exhibit power laws for avalanche size *S* and duration *T* with the following form:

(1) $$P(S)∼S^{-\tau },$$

(2) $$P(T)∼T^{-\alpha }.$$

Therefore, we tested for the presence of power law distributions in neuronal avalanches. To statistically evaluate the presence of power law distributions, we used an importance sampling approach to compare the likelihood of our data being generated by a power-law compared to a log normal distribution, an alternative heavy tailed distribution conventionally used as an alternative hypothesis (Alstott et al., 2014). For a given power law exponent λ, the likelihood is given by

(3) $$\frac{1}{{\left[z(\lambda )\right]}^{M}}\prod_{k=1}^{M}s_{k}^{-\lambda },$$

with observed avalanche sizes or durations defined from *S_1_, S_2_, S_3_...S_M_. z*(*λ*) is the normalisation constant which is defined as

(4) $$z(\lambda )=\sum_{s=a}^{b}s^{-\lambda },$$

where *a* and *b* are the minimum and maximum avalanche values used as the power law cutoffs (*avalanche size: a =* 3, *b* = maximum observed value, *duration: a* = 2, *b* = maximum observed value). The log likelihood (*log L*) is then defined as

(5) $$logL=-Mlog(z)-\lambda \sum_{k}log\left(s_{k}\right).$$

Log likelihoods were calculated across a range of sampled *λ* values, which were weighted by the log probability of observing *λ*_i_ from the prior and proposal distributions. To be precise, the weight *wi* for exponent *λ*_i_ is given by

(6) $$w_{i}=\frac{likelihood\left(data|\lambda_{i}\right)prior\left(\lambda_{i}\right)}{proposal\left(\lambda_{i}\right)}.$$

Marginal likelihoods (ML) were then estimated as the empirical means of all the weights

(7) $$ML=\frac{1}{N}\sum_{k}w_{k}.$$

Finally, the likelihood ratio (LR) was calculated as the ML of a power law minus the ML of a lognormal distribution, with positive values indicating more evidence for a power law distribution. Avalanche power law exponents were estimated as the maximum likelihood of *λ* given the data

### Avalanche Exponent Relations

It is important to note that the presence of power laws alone is insufficient evidence of criticality, as they can arise from non-critical and random mechanisms (Miller, 1957; Stumpf & Porter, 2012; Touboul & Destexhe, 2017). An alternative criterion which may more robustly assess neuronal criticality is the relationship between avalanche exponents α and τ (Friedman et al., 2012; Sethna et al., 2001). At criticality, if equations (1) and (2) hold, there should be a scaling relation between avalanche size *S* and duration *T*, as

(8) $$\langle \text{S}\rangle (T)∼T^{\gamma }.$$.

This relationship exists because critical systems are self-similar, so a single exponent γ can predict the relationship between S and T across all scales of space and time. At criticality the scaling exponent γ is a function of avalanche exponents α and τ, defined as

(9) $$\gamma =\frac{\alpha -1}{\tau -1}.$$

Importantly, this exponent relationship can separate out critical from non-critical systems which generate power law distributions (Fontenele et al., 2019; Touboul & Destexhe, 2017). However, one should note that even exponent relationships can emerge in random systems (Destexhe & Touboul, 2021). Nonetheless, exponent relations offer an adjunct test for criticality alongside the presence of power laws. To test for critical exponent relationships in avalanche data, we use the previously developed deviation from criticality coefficient (DCC) (Ma et al., 2019). DCC is defined as the absolute difference between empirically derived γ, using the slope of 〈S〉 (T), and the predicted γ, calculated from the equation (α - 1)/(τ - 1). The slope of 〈S〉 (T) was estimated by plotting the mean size 〈S〉 against duration T and fitting exponents via linear regression.

### Branching Parameter

To further test for the presence of criticality in our empirical data, we used a metric known as the branching parameter. A branching process describes how an ancestor element can generate descendant elements (Harris, 1963) – e.g. an active neuron activating other neurons. The branching parameter σ captures the expected number of offspring elements from a given ancestor (Corral & Font-Clos, 2012). Interestingly, branching processes undergo a phase transition when σ = 1, at which point avalanches can span all scales. Evidence from brain recordings suggest that *σ* is close to 1 in critical networks (Beggs & Plenz, 2003). σ was estimated as the mean ratio of descendants to ancestors at each time step over all avalanches defined as

(10) $$\sigma =\frac{1}{t}\sum_{n=1}^{t}\frac{descendants}{ancestors\;}.$$

where *t* is the number of avalanche propagation timesteps over all avalanches.

### Correlation Functions

Long range correlations are another key hallmark of critical systems. For example, in lattice-like systems the correlation length is maximal at criticality, although this is not necessarily the case in systems with long range structural connections. The presence of long-range correlations between distant brain areas can give rise to power law scaling of correlation as a function of distance in resting-state activity (Expert et al., 2011; Lombardi et al., 2020; Ponce-Alvarez et al., 2018). Therefore, to further test for criticality we assessed correlation function power law relationships. Specifically, we define the correlation function

(11) $$r(d)∼d^{-\eta },$$

which defines the relationship between the correlation between two time series *r* and the distance between the sources of those time series *d*, where *η* captures the slope of the power law relationship. *r* was assessed as the Pearson’s correlation coefficient between neuron time series’, while *d* was the Euclidean distance in space. To assess the function *r(d*), neuron-neuron paired distances were placed in 200 bins, equally spaced across the range of neuron-neuron distances of all neurons in the brain. *r(d*) was estimated as the mean correlation within each bin. Given that these relationships are not distributions, we cannot perform the likelihood ratio tests described above. Therefore, to assess closeness of fit to power laws we calculated the Euclidean distance between a power law fitted by linear regression and the empirical function.

### Null Models for Critical Statistics

In order to confirm that the observed critical statistics emerge from the spatial and temporal structure of empirical neuronal activity and would not emerge from random signals, we applied a series of null models to our data. The first null model is the *spatial* null, which tests the hypothesis that critical avalanche dynamics emerge from local neighbourhoods of putatively connected neurons. It was generated by randomly shuffling the locations of neurons such that a neurons’ neighbourhood would consist of neurons scattered across the brain rather than nearby neurons. The second null model is the *correlation* null, which tests the hypothesis that critical avalanche dynamics are generated by the correlation structure of neural activity. It was generated by circularly permuting the time series independently for each neuron, such that the correlation across neurons was lost. The third null model is the *autocorrelation null*, which tests the hypothesis that avalanche dynamics are generated by the integration of activity over time within neurons, rather than from shot noise events. It was generated by randomly shuffling the binarised activity of all neurons in a dependent fashion, such that autocorrelation was lost but the cell-cell correlation retained. To compare critical statistics from empirical and null data, 50 nulls were randomly generated for each fish and for each null type, with avalanches and critical statistics calculated as above.

### Network Model Dynamics & Architecture

To probe the microscale changes driving whole brain avalanche dynamics in seizures, we modelled the brain as a network of spiking neurons (Figure 1G). We considered a network of leaky integrate-and-fire neurons with *N* excitatory neurons (*N* = 8990), and *E* external excitatory inputs (*E* = 1000) onto each neuron. The membrane voltage for neuron *i* is defined at subthreshold voltages by the differential equation

(12) $$\tau_{m}\frac{dV_{i}}{dt}=\frac{-\left(V_{i}-V_{r}\right)}{R_{m}}+I_{i},$$

where *V_i_* is the membrane voltage, *T_m_* is the membrane time constant (*T_m_* = 20), *V_r_* is the resting membrane potential (*V_r_* = 0), *R_m_* is the resistance, and *I_i_* is the input current. For voltages beyond the voltage threshold *V_th_* the neuron fires a spike, at which point *V_i_* is reset to *V_r_*.

The input to neuron *i* at time *t* is defined by

(13) $$\begin{array}{l} I_{i}(t)=\sum J_{ij}\delta \left(t-\tau_{d}\right)\\ +\sum U_{ie}\delta (Poisson,\lambda ). \end{array}$$

The first term describes the input from other neurons in the network, where *J_ij_* is the weighted, directed adjacency matrix, between the presynaptic neuron *i* and postsynaptic neuron *j*. A spike from neuron *j* will affect neuron *i* after a synaptic delay *T_d_* = 1, through Dirac’s delta function. The second term describes the input from the external current, where *U* is the weighted adjacency matrix between the presynaptic neuron *i* and the external neuron *e*. External input spikes are modelled as a Poisson point process with rate *λ* = 10 Hz. *U_ie_* for all connections were set to 0.1.

To accurately model the larval zebrafish brain structure, we embedded our model network in a 3-dimensional brain space that recapitulated the structural boundaries, anatomically-defined neuron densities, and the neuron-neuron distance distributions found in the larval zebrafish brain. Specifically, we registered all neuron coordinates across all brains (10 fish, 3 conditions) to a standard space (Tabor et al., 2019). Next, we performed k-means clustering on the spatial locations of all neurons (k = 8990, the mean number of cells across all datasets), with resulting cluster centroids used as network node locations. This enabled the construction of an ‘average’ larval zebrafish brain, which respected the structural boundaries and the spatial neuronal distributions of the brain, at the scale of empirical recordings. To connect neurons in the network we chose to model the connectivity in a scale-invariant network. Network connectivity was defined by the growth and preferential-attachment algorithm (Barabási & Albert, 1999), that has been extended to brain networks (Eguíluz et al., 2005).

### Network Model Fitting

Having defined the network model, we wanted to probe the specific neuronal parameter changes that drive seizure activity. We defined 3 variable parameters in the neuronal network (Figure 1G): 1) *network connectivity* – the number of connections between neurons defined by parameter *m*, 2) *synaptic weights* – the strength of synapses between neurons defined by parameter *r* , and 3) *intrinsic excitability* – the propensity of individual neurons to spike defined by parameter *V_th_*.

*Network connectivity* was varied by altering *m* in the growth and preferential-attachment algorithm, thus changing the number of binary edges between neurons. *Synaptic weights* defines the distributions of edge weights across the network. To define this, we took into account the metabolic constraints imposed on the formation of synapses over long distances (Ahn et al., 2006) – we modelled synaptic weights throughout the network as an exponentially decaying function showing reduced weights as distance increases. To vary synaptic weights smoothly we created a weight function *w*(*d*) which defines synaptic weights as a function of distance, where *w*(*d*) is defined

(14) $$w(d)=i+e^{\left(\frac{-s}{e^{r}}\right)d},$$

where *i* is the initial non-scaled synaptic weight (*i* = 1.2), *s* is the softening parameter that dictates the magnitude of exponential decay for the synaptic weight over distance (*s* = 0.1), *d* is the neuron-neuron distance and *r* defines the strength of the synaptic weights across the network (weak = low *r*, strong = high *r*). This allowed the smooth variation of the weight-distance relationship across all neurons, from a regime in which synaptic weights were weak for most neuron pairs (except neighbouring ones), to one in which synaptic weights were strong across all neuron pairs. Finally, *intrinsic excitability* was varied by changing the spike threshold for each neuron in the leaky-integrate-and-fire model. Using different combinations of these 3 parameters, we could generate model network dynamics for model fitting.

We simulated network dynamics for 4000 time steps. Spiking activity was binned into 10 timestep windows for avalanche estimation. Model avalanche distributions were fit to empirical data generated from concatenated avalanche size distributions from *spontaneous, pre-ictal* and *ictal-onset* datasets (n=9, 400 frames per dataset). Avalanche simulations were run 9 times, and concatenated together to generate fits to empirical data.

In order to assess the closeness of fit between model and empirical data, we designed a cost function which quantified the distance between empirical and model avalanche distributions. We adapted the mean squared error function with a regularisation term, to account for short-tailed distributions that match the candidate distribution over short-medium avalanche sizes, but have empty bins over the longer tail of the distribution. This cost function was defined as

(15) $$\begin{array}{l} Cost\;=MSE\;R\\ MSE=\frac{\sum_{i=1}^{n}{\left(y_{i}-{\hat{y}}_{i}\right)}^{2}}{n-k}\\ R=\alpha e^{\beta 10^{-5}},\; \end{array}$$

where *y_i_* = *P*(*S*) for each avalanche bin, *n* is the number of avalanche distribution bins, *k* is the number of parameters, *β* is the difference in non-empty bin numbers between model and empirical data, and *a* scales the effect of the regularisation (*α* = 0.09). This allowed us to identify the network parameters that most faithfully produced empirically observed avalanche distributions.

### Network Model Response Properties

Certain network response properties are optimal at criticality. In particular, *in vitro* and *in silico* studies have shown that i) the network-mediated separation (NMS) – a network’s ability to discriminate between inputs (Bertschinger & Natschläger, 2004), and ii) the dynamic range (*δ*) – a network’s ability to represent a wide range of inputs (Shew et al., 2009), should be optimised at the critical point. In order to assess the effect of seizure dynamics on critical network response properties, we quantified NMS and *δ* in network models.

To calculate NMS we provided pairs of inputs, *a* and *b* separated by distance *z* onto different network models. For each input, *x* nodes were randomly activated once the network had reached steady state at time *t* (4000 time steps). Here, network activity was binned into 10 time step windows to approximate network outputs. To distinguish between the separation of network activity due to the input responses, as opposed to the intrinsic variability in the network, we define the normalised NMS property as

(16) $$\left\|n_{a}-m_{b}\right\|-∥n-m∥\text{,}$$

where || || denotes the Euclidean distance. *n_a_* and *n* are the binarised state vectors at time *t*, for identical instantiations of a network that received input *a* or received no input, respectively. *m_b_* and *m* are the binarised state vectors at time *t*, for identical instantiations of a network that received input *b* or received no input, respectively. This enabled the normalisation of the distance between two network states following similar inputs *a* and *b*, to the exact distance between those two states as expected in non-perturbed networks.

We estimated *δ* by adapting previous measures (Shew et al., 2009). Here, we provided input to the network by randomly activating *x* neurons across a range of input sizes (*x* range = 5 - 500, stepsize = 10), once the network had reached steady state (4000 time steps). As above, network activity was binned into 10 time step windows to estimate the network response. In order to separate out the network response to a given input from the ongoing network activity, the corresponding output size was calculated as

(17) $$\sum n_{ai}-\sum n_{i},$$

where *n_a_* and *n* are binarised state vectors for identical instantiations of a network that received input *a* or did not receive input at time *t*, respectively. This enabled the separation of active neurons due to the input from active neurons due to the ongoing activity in the network. *δ* is then defined across the range of input sizes as

(18) $$\delta =10log_{10}\left(\frac{S_{\max}}{S_{min}}\right),$$

where *S_max_* and *S_min_* are the input sizes leading to 90^th^ and 10^th^ percentile over the range of output sizes, respectively.

### Stability of Network Dynamics

Another key property of some critical networks, is that the dynamics are neutral, residing between stability and chaos (Haldeman & Beggs, 2005). This means that points in state space maintain their distance over time, a property that is linked to minimal information loss. In order to test whether epileptic seizures disrupt the neutral dynamics expected in critical systems, we calculated the stability of brain dynamics in empirical and model data.

To do this, we approximate the largest Lyapunov exponent (*λ*), which estimates the divergence of nearby trajectories in phase space. One way to do this is to reconstruct the full dynamical system – here we use Takens’ theorem which states that a reconstructed attractor constructed from a variable *Y_0_*, given by *{Y_0_*(*t*), *Y_0_(t - T)*, ..., *Y_0_(t - (E - 1)T)}*, is topologically equivalent to the original dynamical system *{Y_0_(t)*, *Y_1_(t)*, ..., *Y_E-1_(t)}*, where *E* is the dimension of the system and *T* is the time lag (Takens, 1981). We use the first principal component of the data matrix to reconstruct the attractor for each dataset. The embedding dimension *E* was estimated using the false nearest neighbours approach (Kennel et al., 1992). To estimate *T*, one usually finds the *T* that minimises the mutual information between time series. However, given the slow sampling rate of our recordings relative to the kinetics of GCaMP6s, we opted to use *T* = 1.

To estimate *λ* from reconstructed attractors, we firstly locate the nearest neighbour for each point expressed as

(19) $$d_{i}(0)=min\left\|X_{i}-X_{j}\right\|,$$

where *d_i_*(0) is the initial distance between *X_i_* and its nearest neighbour *X_j_*. From this we calculate the change in distance over time for all nearest neighbours along the attractor which for each *t* is

(20) $$\lambda (t)=\frac{1}{t}\frac{1}{M-t}\sum_{t=1}^{M-t}\ln\frac{d_{i}(t)}{d_{i}(0)},$$

where *d_i_*(*t*) is the distance between *X_i_* and *X_j_* at *t*, and *M* is the number of points on the reconstructed manifold (Rosenstein et al., 1993; Sato et al., 1987). The largest Lyapunov exponent is thus the mean separation rate for nearest neighbour points over a given time period.

To estimate stability in our network model, we take advantage of the fact that we have full control of the network. Therefore *λ* can be calculated by perturbing the network and following trajectories over time. We apply small perturbations to the network model once it has reached steady state (4000 time steps), and follow the trajectories over time, where *λ* is

(21) $$\lambda (t)=\frac{1}{t}\ln\left|\frac{d_{i}(t)}{d_{i}(0)}\right|.$$

### Metastable State Estimation

Another key property of critical networks is that the number of states the system can enter into is maximal (Haldeman & Beggs, 2005). In order to assess the effect of seizures on brain state number, we adapt methods developed by Haldeman & Beggs to calculate metastable states. They define a metastable state as a set of state vectors that are more similar to one another than expected in a random system. To identify metastable states in empirical data, we clustered all state vectors together for a given fish using affinity propagation, which does not require cluster number to be defined *a priori* (Frey & Dueck, 2007). Any clusters for which the number of state vectors belonging to that cluster was equal to 1 were removed. To calculate the similarity between state vectors that belong to a cluster, we define

(22) $$\begin{array}{l} Sim\left({\text{Ω}}^{i},{\text{Ω}}^{j}\right)\\ =\frac{\langle {\text{Ω}}^{i},{\text{Ω}}^{j}\rangle }{\langle {\text{Ω}}^{i},{\text{Ω}}^{i}\rangle +\langle {\text{Ω}}^{j},{\text{Ω}}^{j}\rangle -\langle {\text{Ω}}^{i},{\text{Ω}}^{j}\rangle }, \end{array}$$

where Ω is a state vector and < , > denotes the dot product. For clusters to be identified as metastable, they needed to show greater similarity than expected by chance. We estimated the chance level similarity by performing affinity propagation clustering and similarity calculation on a null network using event-count matched shuffling. Any cluster with higher average similarity than the null network was declared a metastable state.

### Eigenspectrum Analysis

In order to test the effect of epileptic seizures on correlations across many neurons, we take advantage of the eigenspectrum function – this captures the variance of the *n*th principal component. Eigenspectra were calculated by performing principal components analysis (PCA) on covariance matrices generated from neuronal activity data. PCA was performed on raw fluorescence traces – here, we reasoned that some of the intrinsic variation in calcium signal might be missed using conventional spike deconvolution methods, which can introduce artefacts (Theis et al., 2016). Therefore, to minimise the risk of artefacts, we performed PCA and eigenspectra analyses using raw fluorescence traces.

The eigenspectrum slope *ϕ* dictates the degree of variance captured by early versus late components, with a high slope indicating that the variance is dominated by early components. If one large, highly correlated subnetwork dominates the dynamics then the point cloud in state space would fall nearly onto a line – only a few components would be needed to capture the variance of the data. Therefore, high multidimensional correlation would cause a steep eigenspectrum slope – as such we use the eigenspectrum slope as a readout of multi-dimensional correlation.

In order to simulate eigenspectra we use a 1-dimensional model introduced by Stringer et al (2019). We define a function *f(x*) which describes the variance of *n* components across *x* samples as

(23) $$\begin{array}{c} f{\left(x\right)}_{2n-1}=\frac{\cos(nx)}{n^{\frac{\phi }{2}}}\\ f{\left(x\right)}_{2n}=\frac{\sin(nx)}{n^{\frac{\phi }{2}}}, \end{array}$$

which gives rise to covariance eigenvalues that follow *n^-ϕ^*. The quantity *n* was uniformly distributed between 0 and 2π. Changing *ϕ* changes the degree of variance captured by earlier versus later components, and thus alters the slope of the eigenspectrum.

### EEG Avalanche Estimation

In order to test whether avalanche dynamics during seizures are conserved across spatial scales, we also calculated avalanche statistics using EEG recordings from human patients. Anonymised intracranial EEG recordings from stereotactically implanted EEG electrodes were selected from the clinical database at Great Ormond Street Hospital. Patients underwent EEG recording for presurgical evaluation of a pharmacoresistant focal epilepsy of presumed focal onset with or without visible lesion on clinical MR imaging. Ethical approval for the use of anonymised data was granted by the UK Health Regulatory Authority (IRAS 229772) and the Joint Research Office at the UCL-Great Ormond Street Institute of Child Health (Project ID 17NP05). 30 patients were selected based on the availability of at least 3 seizures during the EEG recording; as well as artefact-free baseline segments of corresponding duration that were >60 minutes away from ictal recordings. For each patient, data of 3x30 second windows of baseline awake EEG activity, and 3 clinically identified seizures were selected for analysis. Clinical EEG data were recorded at a sampling frequency of 1024Hz. Raw EEG data were re-referenced to an average reference, and filtered to a 1-250Hz broad frequency spectrum using a finite impulse response filter using the window method with a Hamming filter window in the MNE (MEG and EEG analysis and visualization) toolbox in Python (Gramfort et al., 2013)

The signal from each EEG channel was z-scored against the baseline recording of that channel, by subtracting the baseline mean and dividing it by the baseline standard deviation. Data were then binarised by identifying peaks that exceed a peak amplitude threshold parameter *p* (Arviv et al., 2016). Individual peaks across channels were grouped into a single avalanche if they occurred within a short time window *Δt*. Thus, neuronal avalanches in EEG data were defined as sequences of spatially distributed peaks of oscillatory activity.

### Statistical Analysis & Software

D’agostino’s K^2^ test was used to test for normality in data distributions (α = 0.05). Paired t-tests or Wilcoxon signed-rank tests were used to compare *spontaneous, focal* ictal, *generalised* ictal, state transition data, and human EEG datasets in cases of normality, and non-normality respectively (α = 0.05). Independent t-tests or Mann-Whitney U tests were used to compare different network models in cases of normality, and non-normality respectively (α = 0.05). Bonferroni corrections were used to control for false positives due to multiple comparisons.

To compare network response properties across different models we generated 50 simulations for each parameterised model, comparing the means of each simulation across model conditions. To calculate the effect of changing the network topology parameter *m* on network response properties, we parameterised the network to *preictal* levels before increasing *m* in steps to the levels of the *network connectivity* model, while keeping other parameters fixed. We performed 50 simulations for each *m*. We calculated the correlation between *m* and network response properties using Pearson’s correlation coefficient.

Data was analysed using custom code written in Python. Image registration and cell segmentation was performed using suite2p (Pachitariu et al., 2017). Neural network simulations were run using Brian2 (Stimberg et al., 2019). Statistical hypothesis tests were performed using scipy. Graphs were generated using matplotlib and seaborn.

### Resource Accessibility

Details of key reagents and resources are included in Table 1. Further information, requests for resources and datasets should be directed to the corresponding author, Richard Rosch (*richard@dynamic-brains.com*). *Data will be made accessible at* https://osf.io/em7f2/

### Code Accessibility

Custom written python code can be accessed at: https://github.com/dmnburrows/criticality https://github.com/dmnburrows/avalanche_model https://github.com/dmnburrows/seizure_dynamics https://github.com/dmnburrows/img_process

## Results

In this study we recorded single neuron activity brain-wide, to measure critical dynamics during epileptic seizures. We performed 2-photon imaging of head-fixed larval zebrafish across 10 planes (Figure 1A,B), using GCaMP6s expressed only in the nucleus, which enables the segmentation of ~10,000 neurons per animal (Figure 1C). To image seizure dynamics we administered *5mM PTZ* causing focal hyper-excitability (Movie 2), followed by *20mM PTZ* causing generalised hyper-excitability (Movie 3 & Figure 1E). The on/off states of each neuron were then estimated using a hidden Markov model (HMM) (Diana et al., 2019) (Figure 1E). From these data we were able to calculate the spatiotemporal propagation of activity through the network (see Methods & Figure 1F), known as neuronal avalanches (Beggs & Plenz, 2003). Importantly, certain features of avalanche dynamics are found in systems at criticality. We use these features to ask whether neuronal avalanche dynamics across the whole brain deviate from criticality during seizures.

### Spontaneous Brain-Wide Neuronal Activity Exhibits Critical Statistics

Several statistical features have been reported as indicators of criticality, including (i) power law distributions of avalanche size and duration, (ii) relations between avalanche power law exponents, (iii) branching ratios close to unity and (iv) power law scaling of neuron correlation and distance. We first evaluate each of these features to validate the presence of critical dynamics in brain-wide networks at single cell resolution during spontaneous activity.

**Figure 3:**
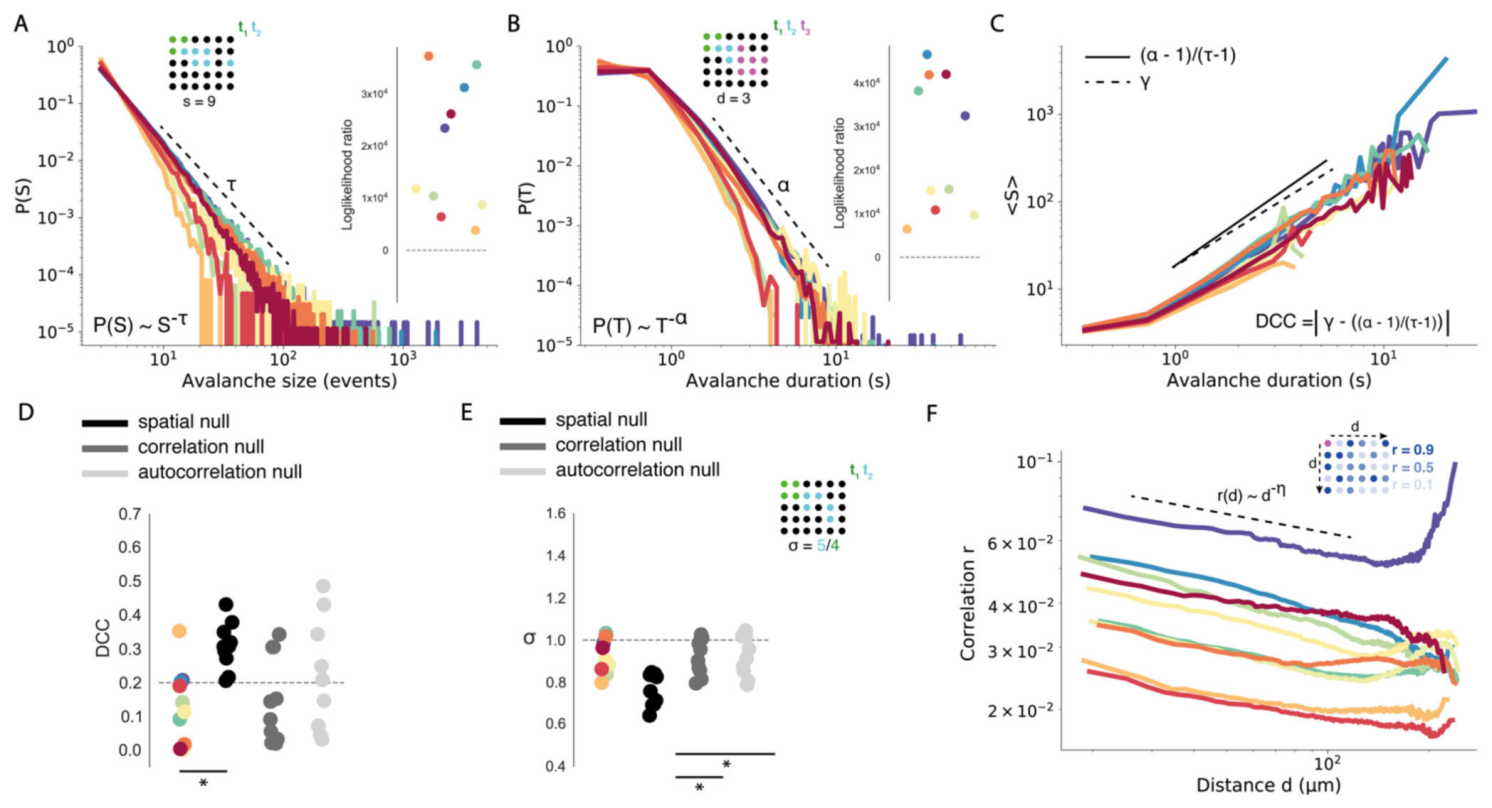
Whole brain spontaneous neuronal activity exhibits critical statistics. (A) Empirical distributions for avalanche size (*S*) with dotted line showing power law exponent *T*, and corresponding log likelihood ratio tests for power law versus lognormal distributions (right). Data points are coloured by fish. Avalanche schematic demonstrating the calculation of avalanche size *s* (top) for a single avalanche event, where coloured dots represent active neurons at *t_x_*. (B) Same as in (A), for avalanche duration (*T*) with exponent *α*. (C) The scaling relationship between *S* and *T* is shown by plotting mean *S* against *T* and fitting a linear regression line to estimate the exponent γ (dotted line). The exponent relation (α-1)/(τ-1) is calculated using avalanche exponents for *S* and *T*. DCC is calculated as the absolute difference between γ and (α-1)/(τ-1). (D) DCCs plotted for each fish against each null model (dotted line = critical threshold of DCC < 0.2). (E) Branching ratio σ plotted for each fish against each null model (dotted line = critical value of σ~1). Avalanche schematic demonstrating calculation of σ (right). (F) The quantity *r(d*), which estimates pairwise neuronal correlation as a function of distance, follows an approximate power-law with exponent *η* (dotted-line). Schematic demonstrating estimation of correlation (*r*) as a function of distance (*d*) (right). Magenta neuron is the neuron of interest. Other neurons are coloured by their correlation *r* to magenta neuron. * = p<0.01

**Figure 4:**
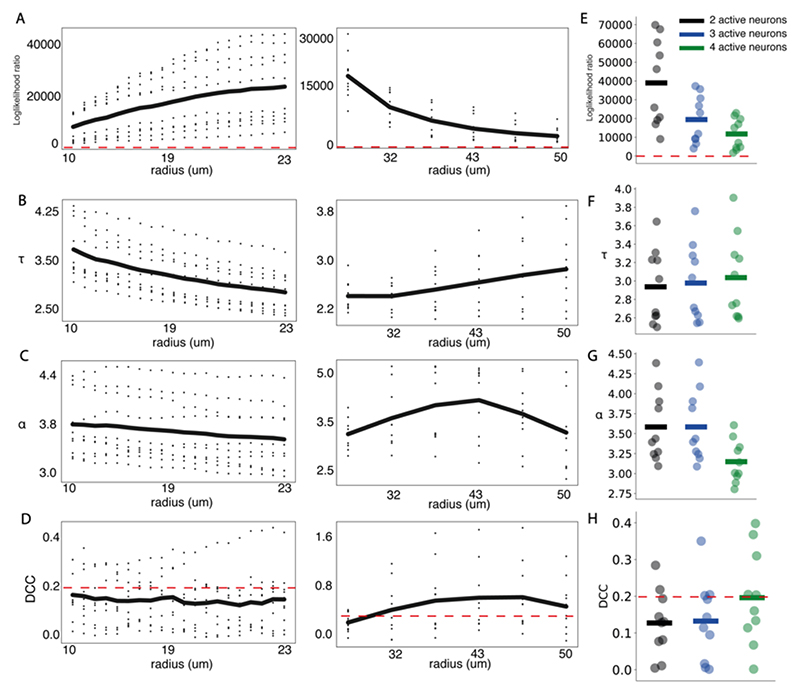
Effect of avalanche parameters on critical statistics. (A-D) Criticality statistics in spontaneous activity for increasing avalanche neighbourhood size *k*, over smaller (left, k = 0.04 – 0.23%; 10 – 19um) and larger ranges (right, k = 0.5 – 3.0%; 23 – 50um). (E-H) Criticality statistics in spontaneous activity for different minimum avalanche initiation sizes, from 2-4.

A key feature of criticality is the presence of scale-invariant avalanche dynamics – these describe the spatio-temporal propagation of activity through the network (Figure 2). At criticality avalanches span all scales, from small to large, which gives rise to scale-invariant power law distributions of avalanche size and duration (Hesse & Gross, 2014; Sethna et al., 2001). To calculate avalanche dynamics, we adapted an algorithm used in previous studies to single neuron data (see Methods) (Ponce-Alvarez et al., 2018). We estimated empirical distributions for avalanche size and duration from spontaneous activity, which were well fit by power laws (Figure 3A,B). Using log likelihood ratio testing, we found that all datasets were better explained by power law than lognormal distributions, the most rigorous alternative heavy-tail distribution test (see Methods & Figure 3A,B). Importantly, we found that the presence of power law relationships was robust to avalanche estimation parameters. Specifically, we varied the neighbourhood radius (from 10 to 50um), which defines the radius of neighbours that a given neuron can send outputs to, and the number active neurons required for avalanche initiation (from 2-4) (see Methods). Avalanche distributions were power laws across all sampled parameter values (Figure 4A,E), suggesting that the presence of power laws in avalanche distributions is a feature of the underlying data, rather than an artefact of the avalanche estimation algorithm. This indicates that avalanche dynamics in spontaneous activity are scale invariant, as expected at criticality.

However, power laws can also emerge in non-critical systems. An adjunct test for criticality is the relationship between the power law exponents for avalanche size (τ) and duration (α) (Friedman et al., 2012; Sethna et al., 2001). At criticality, avalanche size S should scale as a power of avalanche duration T – this scaling exponent γ should follow the form (α-1)/(τ-1) (see Methods). We use the deviation from criticality coefficient (DCC, see Methods) to assess exponent relation, which compares the exponent γ of the function 〈S〉 (T) with the exponent relation (α-1)/(τ-1) (Figure 3C) (Ma et al., 2019). Our data show close agreement between the slope γ and (α-1)/(τ-1), suggesting the presence of near-critical dynamics (DCC = 0.13 ± 0.04) (Figure 3D). To understand whether such relationships could have been generated by a random system, we created 3 null models: *spatial, correlation* and *autocorrelation* nulls (see Methods). With these models we were able to assess whether randomly generated neuronal activity, resulting from shuffling spatial structure, cell-cell correlation and autocorrelation respectively, would also generate critical DCCs. Importantly, exponent relation was significantly less well preserved in *spatial* nulls (DCC = 0.31 ± 0.02, t = -4.80, p < 0.001) compared to empirical data, but not *correlation* (DCC = 0.15 ± 0.04, t = - 0.48, p = 0.64) and *autocorrelation* (DCC = 0.21 ± 0.05, t = -1.60, p = 0.15) nulls (see Figure 3D). This suggests that empirically observed critical exponent relations emerge due to the spatial structure of neural dynamics, rather than from random activity. This provides further evidence for critical statistics in brain-wide cellular resolution network dynamics at rest.

Another hallmark of critical systems is abranching parameter (σ) close to 1 (see Methods). The branching parameter describes the expected number of offsprings that an ancestor produces, and when σ~1 the system undergoes a phase transition and avalanches can span all scales (Corral & Font-Clos, 2012). We calculate σ by estimating the mean ratio of descendants to ancestors in spontaneous data (see Methods), and find σ slightly below 1 in spontaneous activity (σ = 0.93 ± 0.03) (Figure 3E). Values of σ slightly below 1 suggest the presence of a slightly subcritical state (Priesemann et al., 2014), however σ is likely to be underestimated in finite networks due to the convergence of activity onto shared descendants (Zierenberg et al., 2020). We complement our approach with an alternative method which uses multiple-regression and is robust to sub-sampling to confirm σ values (Spitzner et al., 2021; Wilting & Priesemann, 2018). Using this approach we find σ even closer to 1 (σ = 0.97 ± 0.00), providing further evidence for near-critical dynamics in spontaneous activity. Importantly, using our method, we find that empirical σ is significantly closer to unity than *spatial* (σ = 0.77 ± 0.03, t = 21.8, p <0.0001), and *correlation* (σ = 0.92 ± 0.03, t = 4.45, p <0.01), but not *autocorrelation* nulls (σ = 0.93 ± 0.03, t = - 0.28, p = 0.79) (Figure 3E). This suggests that σ near to 1 in our data is a feature of the underlying spatial structure and correlation in neural activity, suggesting near-critical dynamics in our spontaneous activity data.

Long-range correlations between neuronal signals are another hallmark of critical systems. Such long-range correlations can give rise to a power law relationship between distance and correlation in spontaneous activity at criticality (Expert et al., 2011; Lombardi et al., 2020). To assess the presence of correlation-distance power laws, we calculated the correlation function *r(d*) as the Pearson correlation between the activity of neuron pairs as a function of their distance (see Methods and Figure 3F). Interestingly, we found that *r(d*) qualitatively follows a power law which is well approximated by linear regression fit (Figure 3F). This suggests the presence of both short and long-range correlations which can support avalanches spanning the full spatial scale of the system.

Taken together we find that spontaneous neuronal dynamics exhibit (i) power law relationships of neuronal avalanches, (ii) exponent relations close to critical values, (iii) branching ratios close to 1, and (iv) power law scaling of neuron correlation and distance. These findings provide evidence that microscale collective behaviour is organised near to criticality in whole brain networks.

### Brain-Wide Neuronal Activity Deviates from Criticality During Seizures

In a next step, we investigated whether whole brain networks at the microscale deviate from criticality during epileptic seizures. To this end, we compared critical statistics in spontaneous activity with focal (*5mM PTZ*) and generalised seizure (*20mM PTZ*) dynamics.

**Figure 5:**
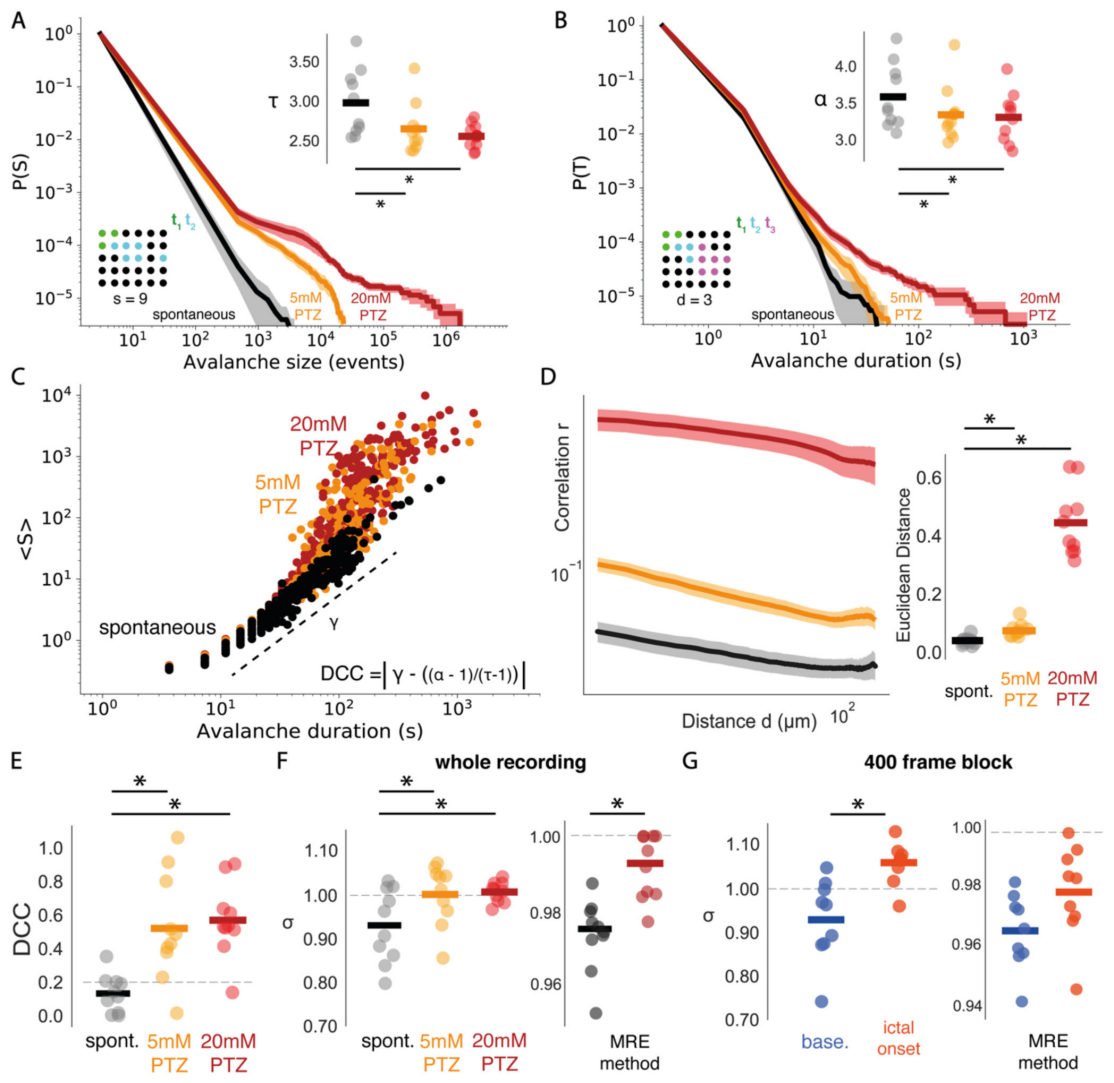
Seizures cause a loss of whole brain critical statistics. (A) Complementary cumulative distributions for avalanche size (*S*), showing mean distributions across conditions (shaded regions represent the standard error), with power law exponents *T* compared (top right) across *spontaneous* (black)*, 5mM PTZ* (orange) and *20mM PTZ* (red) conditions. (B) Same as in (A), for avalanche duration (*T*) with exponent *α*. (C) The scaling relationship between *S* and *T* is shown by plotting mean *S* against *T* and fitting a linear regression line to estimate the exponent γ (dotted line), as shown for *spontaneous* (black)*, 5mM PTZ* (orange) and *20mM PTZ* (red) conditions. DCC is calculated as the absolute difference between γ and (α-1)/(τ-1). (D) The correlation function *r(d*) compared across conditions (left), with the Euclidean distance from fitted power laws plotted for each condition (right). (E) DCC compared across conditions. (F) The branching parameter σ plotted for each dataset across 30 minute recordings, using avalanche (left) and multiple-regression estimation approaches (right). (G) Same as in (F), for shorter 400 frame state transition periods, where *baseline (base.*) is before seizure transition and *ictal onset* is immediately following transition. * = p<0.01

We first compared avalanche exponents, estimated from the full range of observed avalanche sizes and durations (see Methods), to determine if seizures alter avalanche dynamics. Interestingly, avalanche exponents for both size and duration significantly decreased, in the *5mM* (Size: T = 2.65 ± 0.10, w = 0, p < 0.01; Duration: α = 3.34 ± 0.12, w = 2.0, p < 0.01) and *20mM* conditions (Size: T = 2.56 ± 0.05, t = 4.59, p < 0.01; Duration: α = 3.30 ± 0.11, t = 4.06, p < 0.01) when compared with spontaneous activity (Size: T = 2.98 ± 0.13; Duration: α = 3.58 ± 0.14) (Figures 5A,B). This manifested as a less steep slope during focal and generalised seizures, indicating a higher probability of larger and longer avalanches. Furthermore, the *20mM PTZ* condition caused a bump in the heavy tail of the distribution (Figures 5A,B), suggesting the emergence of characteristic scale due to the occurrence of unusually common, excessively large and long avalanches, which is expected in ‘supercritical’ systems driven away from criticality (Beggs & Plenz, 2003).

**Figure 6:**
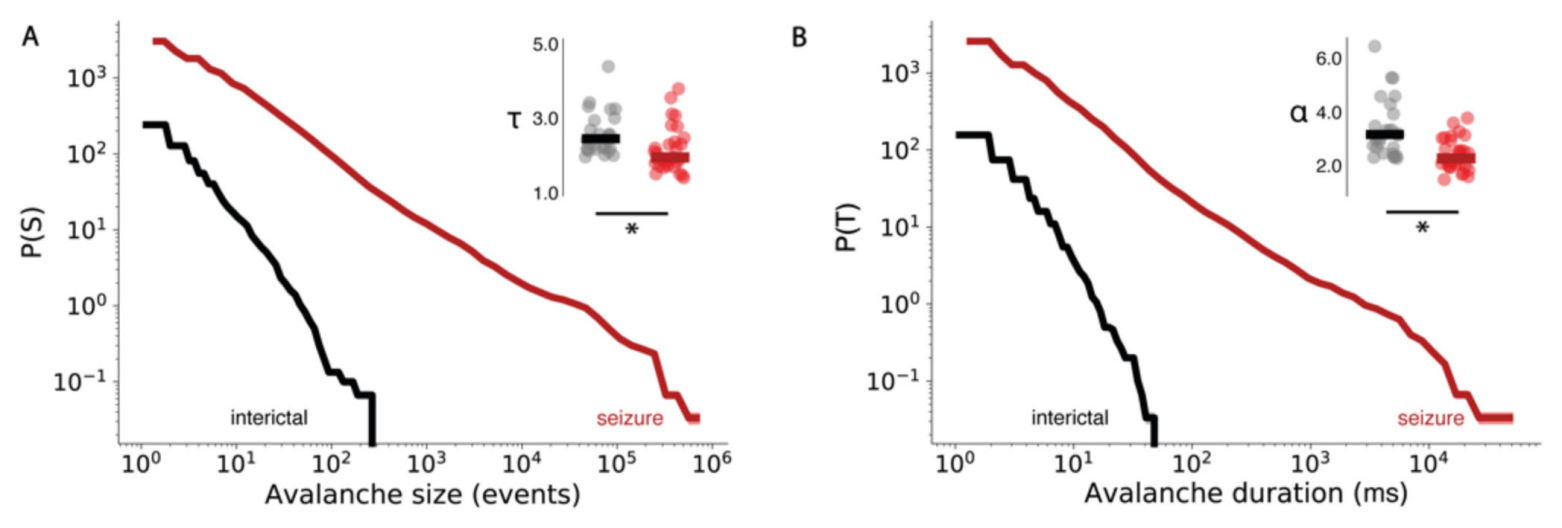
Human epileptic seizures at the macroscale alter avalanche dynamics. Complementary cumulative distribution functions for avalanche size (A) and duration (B), comparing mean distributions across EEG datasets for inter-ictal (black) and seizure periods (red). Avalanche exponents are compared for avalanche size ( and duration (α) in *interictal (* black bar = mean), and *seizure* (red bar = mean) conditions.

Next, we investigated whether such alterations to avalanche probabilities were conserved across brain scales. Specifically, various studies have assessed avalanche dynamics at coarse scales using EEG and MEG (Arviv et al., 2016; Meisel et al., 2012), however whether such macroscale avalanche dynamics capture the underlying collective behaviour of neuronal activity is unclear. To this end, we calculated avalanche dynamics from sequences of oscillatory peaks in human intracranial EEG recordings during seizures (see Methods), to ascertain whether larger and longer avalanches are also recorded at the macroscale. We found that focal-onset seizures in epilepsy patients gave rise to concordant decreases in avalanche exponents (Size: median T = 1.94, 1.38-3.92, U = 3.71, p < 0.001; Duration: median α = 2.25, 1.47-3.77, U = 4.24, p < 0.001), when compared with inter-ictal periods (Size: median T = 2.52, 1.99-5.9; Duration: median α = 3.16, 2.26-11.64) (Figure 6). This suggests that the presence of longer and larger avalanches at the microscale during seizures, is also conserved in macroscale EEG data. However, we note that specific avalanche exponents are not conserved across scales, suggesting intrinsic differences between power law behaviour at each scale.

Next, we assessed the effect of PTZ-induced seizures on critical exponent relations. Interestingly, upon visual inspection, while the relationship between *S* and *T* clearly follows a power law in spontaneous datasets, this relationship appears to be lost during seizures (Figure 5C). Specifically, while small avalanches in PTZ conditions appear to follow the scaling relationship γ defined from spontaneous activity, as avalanches get larger in size they occur at a faster rate than expected from the scaling exponent γ. This suggests a loss of self-similarity in avalanche dynamics during seizures, which is a defining feature of criticality (see Methods). A loss of power law scaling between S and T implies that the critical exponent relationship γ= (α-1)/(τ-1) would break down (Perković et al., 1995). To confirm this we assessed DCC values – as expected seizures caused a divergence from critical exponent relations, with both *5mM PTZ* (DCC = 0.52 ± 0.10, t = -3.24, p = 0.01) and *20mM PTZ* (DCC = 0.57 ± 0.07, t = -5.70, p < 0.001) causing significant increases in DCCs (Figure 5E). Therefore, seizures disrupt the collective behaviour of neuronal activity at the microscale such that self-similarity and critical exponent relations are lost.

**Figure 7:**
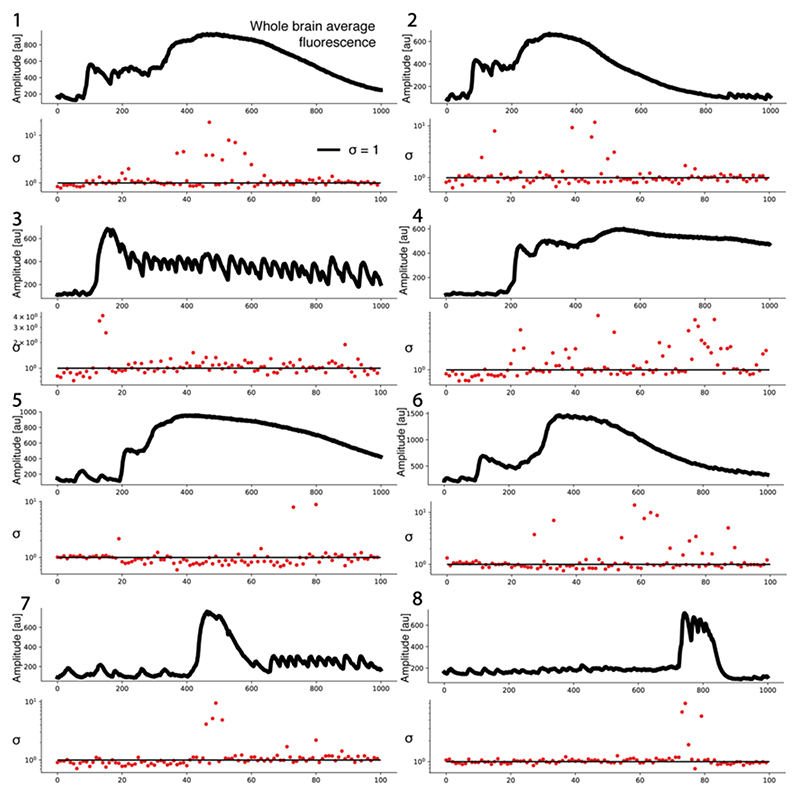
Branching parameter assessed throughout seizure evolution. The branching parameter σ was estimated using the avalanche approach across 1000 frames of generalised seizure periods in 10 frame bins. 2 datasets were excluded based on the absence of clear transitions from non-generalised to generalised activity, or insufficient frames following the transition initiation. σ remains close to 1 in the leadup to the generalised seizure and drastically increases as dynamics become highly synchronous, for most datasets (2, 3, 4, 5, 7, 8). Interestingly, following initial increases, σ quickly returns to ~ 1 while high synchrony is ongoing. Note that for some datasets (1, 6) σ remains close to 1 until after the onset of highly synchronous activity. Oscillatory dynamics following the peak of synchronous activity are characterised by moderate increases in σ above 1 (3, 7).

We also assessed the effect of seizures on the branching parameter σ which is expected to be close to 1 at criticality. We found a significant increase in both the *5mM PTZ* (σ = 1.00 ± 0.02, t = -3.79, p <0.01) and *20mM PTZ* conditions (σ = 1.01 ± 0.01, t = -3.11, p < 0.017), suggesting an increased propensity for a neuron to activate descendants (Figure 5F). To confirm this change, we also calculated σ under seizure conditions using the multiple-regression approach – here we also find a significant increase in the branching parameter during seizures (*20mM PTZ*, σ = 0.99 ± 0.00, t = -5.22, p < 0.0001) (Figure 5F). Interestingly, these changes indicate that brain dynamics may be operating closer to criticality during seizures as σ approaches 1. However, identifying σ > 1 is difficult in finite-size networks as avalanches will always be bounded by the size of the system (Zierenberg et al., 2020). One approach to identify σ > 1 in finite systems, is to look at σ over shorter periods, to capture the extending phase of the avalanche before it is reaches the full size of the system (Hagemann et al., 2021). To this end we also compared σ during shorter 400 frame windows of spontaneous activity (*baseline*) and generalised seizure (*ictal-onset*) (see Methods). Using our method, we found that σ shows a greater magnitude increase beyond 1 during seizure onset (*baseline*: σ = 0.94 ± 0.03, *ictal-onset*: σ = 1.05 ± 0.02, t = -3.41, p <0.01) (Figure 5G). To confirm this finding, we also calculated the branching parameter in short 10 frame bins throughout generalised seizure transitions. As expected, σ drastically increased beyond 1 over short periods across all tested datasets, indicating transient departures from criticality (Figure 7). Therefore, seizures transiently cause an increase in the branching parameter above 1, as expected for a supercritical system pushed away from criticality. Interestingly, σ dynamics were highly diverse across seizure periods, with mixtures of critical (σ~1) and supercritical (σ >1) branching values throughout seizure evolution (Figure 7).

However, we note that using the multiple regression approach on 400 frame datasets led to a non-significant increase in σ (*baseline*: σ = 0.96 ± 0.00, *ictal-onset*: σ = 0.98 ± 0.01, w = 9.0, p =0.13) (Figure 5G). Taken together, the direction of the branching parameter change relative to the phase transition is unclear from our data.

Finally, we assessed the effect of seizures on correlation function power laws. Interestingly, correlation functions were significantly less well approximated by power laws in both the *5mM* (Euclidean distance from power-law = 0.07 ± 0.01, w = 1.0, p <0.01) and *20mm PTZ* conditions (Euclidean distance = 0.44 ± 0.04, w= 0.0, p <0.01) compared with spontaneous datasets (Euclidean distance = 0.04 ± 0.00) (see Methods & Figures 5D). This indicates that seizures saturate the system with excessive correlations, thus disrupting power law relationships that are a hallmark of criticality.

Taken together, we find that seizures cause a loss of critical statistics, with i) the emergence of characteristic scale in avalanche distributions, ii) a departure from critical exponent relations, iii) increases in near-critical σ values, and iv) a loss of correlation function power laws. That avalanches sampled at single neuron resolution across the whole brain, exhibit a loss of critical statistics during seizures, indicates that microscale neuronal activity collectively drives network-wide dynamics away from the phase transition during seizures. Next, we wanted to understand what microscale network changes facilitate this loss of criticality during seizures.

### Densely Connected Networks Drive Avalanche Dynamics Away from Criticality

If seizures emerge as a loss of criticality, what network changes drive the brain away from the phase transition into seizure states? Interestingly, synaptic genes are risk factors in epilepsy (Rosch et al., 2019) and synaptic plasticity is essential for organising critical dynamics *in silico* (Zeraati et al., 2021). As network models have demonstrated a diversity of synaptic mechanisms supporting self-organising criticality, these approaches may provide clues into the synaptic pathways which, when dysregulated in epilepsy, give rise to a loss of criticality and seizures. In particular, modelling studies have implicated the number of connections in the network (Tetzlaff et al., 2010), the strength of synapses (de Arcangelis et al., 2006) and the intrinsic excitability of neurons (Levina et al., 2007), in stabilising brain dynamics to criticality.

**Figure 8:**
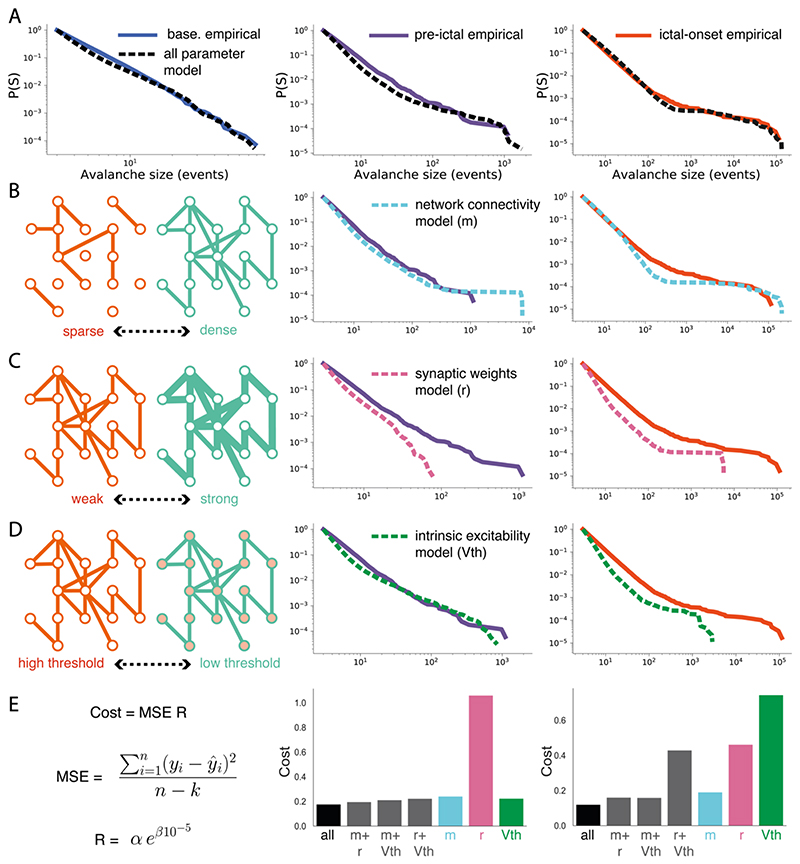
Neuronal network mechanisms driving generalised seizure dynamics. (A) Avalanche distributions from *baseline, preictal* and *ictal-onset* periods, alongside best model fits using all parameters. (B) Network connectivity schematic for sparse and dense networks (left). Best model fits using network connectivity parameter only, to *pre-ictal* (middle) and *ictal-onset* data (right). (C) Synaptic weights schematic for strong and weak synaptic weights (edge thickness = synaptic strength) (left). Best model fits using synaptic weights parameter only, to *pre-ictal* (middle) and *ictal-onset* data (right). (D) Intrinsic excitability schematic for high and low threshold to spike networks (red node = closer to spike) (left). Best model fits using intrinsic excitability parameter only to *pre-ictal* (middle) and *ictal-onset* data (right). (E) Model comparison for 3 parameter models (black), 2 parameter models (grey) and single parameter models (colours as above), for *pre-ictal* (middle) and *ictal-onset* data. The cost function used for model comparison was a regularised mean squared error term (left, See Methods).

To probe the synaptic changes that drove avalanche dynamics away from criticality into seizure states, we modelled the brain as a spiking network of excitatory leaky-integrate-and-fire neurons (see Methods). We were particularly interested in the network mechanisms supporting the emergence of seizure state transitions. Accordingly, we fit network models to 3 avalanche datasets comprising normal activity (*baseline*), activity immediately preceding the generalised seizure (*pre-ictal*), and the generalised seizure itself (*ictal-onset*) (see Methods). To distinguish between the differential involvement of synaptic changes in driving seizure state transitions, we investigated 3 parameters in our model: 1) *network connectivity* – the number of connections between neurons defined by parameter *m*, 2) *synaptic weights* – the strength of synapses between neurons defined by parameter *r*, and 3) *intrinsic excitability* – the propensity of individual neurons to spike defined by parameter *V_th_* (Figure 8). While a diversity of other parameters can drive epileptic seizures, including neuronal subtype (Khoshkhoo et al., 2017; Magloire et al., 2019; Sessolo et al., 2015), non-synaptic (Bikson et al., 2018; Dudek et al., 1998; Magloire et al., 2022) and non-neuronal mechanisms (Coulter & Steinhäuser, 2015; Diaz Verdugo et al., 2019; Vezzani et al., 2022; Wu et al., 2020), we restricted our model to 3 simplified plasticity parameters, in order to relate predictions from criticality theory with observed seizure avalanche dynamics. Importantly, such simplified plasticity models make few assumptions about the underlying dynamics, and if they can explain observed data, can demonstrate the sufficiency of general synaptic mechanisms in driving seizure activity and critical dynamics.

We performed a grid-search of ~1,400 parameter combinations, finding the combinations which best captured *baseline, pre-ictal* and *ictal-onset* avalanche dynamics. Using all 3 parameters, we generated approximate model fits to *baseline* (m = 7, r = 5, V_th_ = 20, cost = 0.113), *pre-ictal* (m = 6, r = 0, V_th_ = 16, cost = 0.176), and *ictal-onset* data (m = 31, r = 1, V_th_ = 17, cost = 0.120) (Figure 8A).

To compare the importance of each network parameter in the emergence of the *pre-ictal* state, we explored a subset of parameters that were free to vary, while keeping others fixed. Specifically, we fixed parameters to the best *baseline* model fit while allowing subsets of parameters to vary freely for model fitting. This was done because the partially hyper-excitable dynamics of the *pre-ictal* state were preceded by a baseline activity state before the addition of PTZ. Allowing only single parameters to vary freely for fitting demonstrated that the *intrinsic excitability* parameter provided the best fit to *pre-ictal* data (*V_th_* = 19, cost = 0.223) (Figure 8D), through increases in intrinsic excitability. *Network connectivity* changes alone gave rise to avalanche distributions with excessive heavy tails (*m* = 15, cost = 0.241) (Figure 8B), while *synaptic weights* changes alone were unable to generate sufficiently heavy tails (*r* = 6, cost = 1.06) (Figure 8C). However, we found that the model with all 3 parameters free to vary provided the best *pre-ictal* fit (Figure 8E), while models with any combination of 2 free parameters provide better fits than *intrinsic excitability* alone. Therefore, while increases in intrinsic excitability is the singular parameter change that best explains the emergence of pre-ictal dynamics, non-specific combinations of all three parameters can more accurately describe such dynamics. This indicates that the *pre-ictal* state likely emerges from small, non-specific changes to *network connectivity*, *synaptic weights* and *intrinsic excitability*.

Next, to model the emergence of the *ictal-onset* state, we fixed parameters to the best *pre-ictal* model fit while allowing subsets of parameters to vary freely for model fitting. Allowing only single parameters to vary demonstrated that only the *network connectivity* parameter provided a reasonable fit to *ictal-onset* data (*m* = 30, cost = 0.190) (Figure 8B), emerging due to an increase in the number of connections from the *pre-ictal* state. Changing both *synaptic weights* (*r* = 7, cost = 0.465) and *intrinsic excitability* (*V_th_* = 15, cost = 0.744) parameters alone failed to generate sufficiently heavy tails (Figure 8C,D). This suggests that increases in network connectivity are central to driving generalised seizure dynamics and thus pushing the brain away from criticality. In fact, while model fits with all 3 parameters provided the best overall fit, the *network connectivity* model alone provided a better fit than the model with both *synaptic weights* and *intrinsic excitability* free to vary (Figure 8E). Therefore, increasing the connectivity of the network alone is sufficient to drive the heavy-tailed avalanche dynamics of generalised seizures.

Taken together, we find that the emergence of the pre-ictal state is driven by non-specific changes to combinations of neuronal connectivity, synapse strength and node excitability. Conversely, generalised seizure dynamics can only emerge in densely connected networks. This indicates that the density of network connections is a key parameter for disrupting critical dynamics during seizures. Next, we investigated the effects of such dense connectivity on the emergent computational properties of critical networks.

### Dense Connectivity Drives the Emergence of Chaos in Seizure Networks

Having identified key neuronal parameters driving generalised seizure dynamics, we next investigated the functional implications of such changes. Interestingly, during generalised seizures patients regularly exhibit a complete loss of awareness (Bikson et al., 2018; Dudek et al., 1998; Magloire et al., 2022; Shimoda et al., 2020). Given that generalised seizures emerge as a loss of criticality, we theorised that co-occuring cognitive dysfunction would be caused by the suboptimal computational capacities of networks away from criticality. One key prediction for critical networks is that the phase transition can separate out stable and chaotic regimes, where the critical point exhibits neutral dynamics (Haldeman & Beggs, 2005). Such neutral dynamics give rise to long autocorrelation and minimal information loss about inputs (Hesse & Gross, 2014). Therefore, if critical brain dynamics exist at the boundary between stability and chaos, then one would expect a loss of neutral dynamics during generalised seizures.

**Figure 9:**
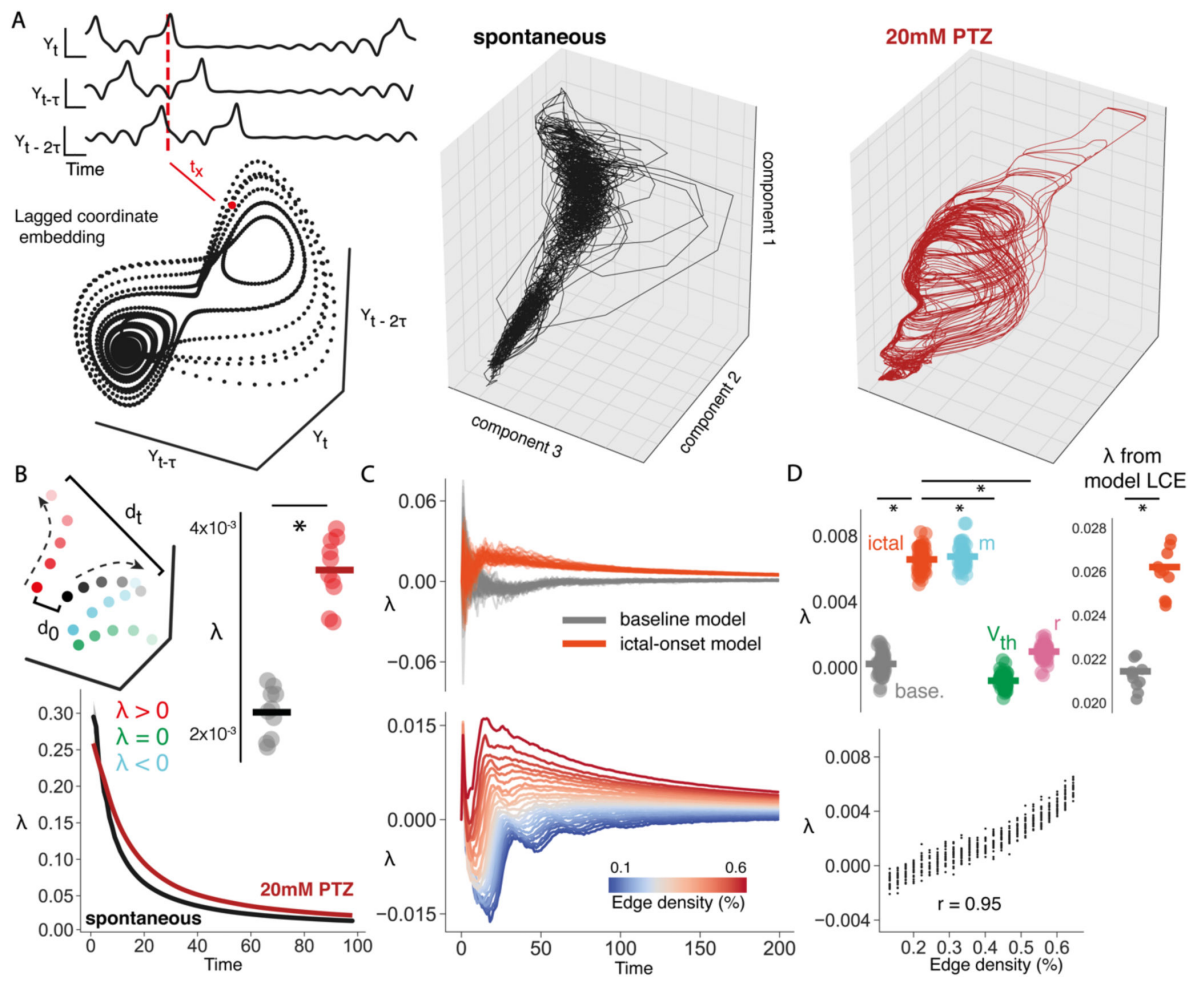
Increased connectivity drives the emergence of chaos in seizure networks. (A) Using lagged coordinate embedding single variable (*Y*) can be used to reconstruct an attractor that is topologically equivalent to the full system, by using a series of delayed variables (*Y, Y_t-T_, Y_t-2T_ …, Y_t-(m-1)T_*) of delay *T* and dimension *m*. Embedding each lagged variable into state space provides the reconstructed attractor, where *t_x_* is the position in state space at time *x* corresponding to the dotted red line (left). Isomap 3d embeddings of reconstructed attractors using lagged coordinate embedding for spontaneous (middle) and 20mM PTZ (right) data for a representative fish. (B) Schematic outlining the meaning of different *λ* values (top). Each colour represents the trajectory over time for a specific initial point along the attractor (high to low brightness represents movement in time). *λ* is the ratio of the difference between 2 points at the start (d0) and at *t* (dt). *λ* > 1: chaotic (red), *λ* < 1: stable (blue), *λ* = 1: neutral (green), where *λ* for each trajectory is calculated against the black trajectory. *λ* over time is compared for *spontaneous* and *20mM PTZ* conditions (bottom), and mean *λ* is compared across *spontaneous* (black bar = mean) and *20mM PTZ* conditions (red bar = mean) (top right). (C) *λ* over time compared for *baseline* and *ictal-onset* full parameter models (top), and *λ* over time is shown as a function of edge density *m* (bottom), ranging from *pre-ictal* values to *ictal-onset* values. Each line represents the mean *λ* over time for each model over 50 simulations. (D) Mean *λ* compared for *baseline (base.*) and *ictal-onset* full parameter models, and single parameter models (*m = network connectivity, Vth = intrinsic excitability, r=synaptic strength*) (top left). Correlation between *m* and mean *λ* is shown, ranging from *pre-ictal* values to *ictal-onset* values (bottom). Mean *λ* calculated using lagged coordinate embedding (LCE) on full parameter models across *baseline* (grey bar = mean) and *ictal-onset* models (crimson bar = mean) (top right).

To test this hypothesis, we approximated the largest Lyapunov exponent (*λ*). *λ* captures the distance of nearby trajectories in state space, where *λ* > 0 implies that points grow further apart over time (chaos), *λ* < 0 implies that points get closer over time (stable), and *λ* ~ 0 implies that points retain their distances over time (neutral) (Figure 9B). *λ* was estimated from *spontaneous* and *20mM PTZ* empirical datasets, by reconstructing the attractor of the system using Takens’ embedding theorem (see Methods & Figure 9A). We found that generalised seizures were significantly more chaotic (*20mM PTZ:* λ = 0.0034 ± 0.0001, t = -10.7, p <0.0001), than *spontaneous* dynamics (*λ* = 0.0024 ± 0.0001) (Figure 9B). To confirm this finding, we also calculated λ in our model network, by directly perturbing the system and following the trajectories over time (see Methods). Using this approach we find that *ictal-onset* models were also significantly more chaotic than *baseline* models (*baseline: λ* = 0.0002 ± 0.0001, *ictal-onset:*λ= 0.0065 ± 0.0001, t = -45.5, p < 0.0001), using all-parameter models (Figure 9C,D). This indicates that close-by trajectories will grow further apart over time, suggesting a heightened sensitivity to initial conditions in the seizure state (Figure 7B).

To confirm the validity of our embedding approach we also compared model *λ* as generated from the direct perturbation and embedding approaches (see Methods). Importantly, the embedding approach also shows a significant increase in *λ* in the *ictal-onset* period in the model (*baseline:*λ= 0.02 ± 0.0003, *ictal-onset:*λ= 0.03 ± 0.0004, U = 0.0, p < 0.0001) (Figure 9D). Therefore this indicates that during generalised seizures the brain enters a chaotic state, as expected for a supercritical network away from criticality (Haldeman & Beggs, 2005).

Next, to relate observed chaotic dynamics with underlying neuronal parameter changes driving seizures, we tested the link between *λ* and *network connectivity* in the network model. To do this we compared *λ* across single parameter models, with fixed parameters set to *pre-ictal* values and free parameters fitted to *ictal-onset* data. Interestingly, only the *network connectivity m* model produced λ that matched the full model (0.0067 ± 0.0001, U = 1108.0, p = 0.16), while *synaptic weights r* (0.0010 ± 0.0001, U = 0.0, p < 0.0001) and *intrinsic excitability Vth* (-0.0010 ± 0.0001, U = 0.0, p < 0.0001) models failed to produce more chaotic dynamics (Figure 9D). This suggests that only the *network connectivity* parameter can drive the emergence of chaotic dynamics observed during generalised seizures. We also found a significant positive correlation between the number of connections *m* and *λ* (r = 0.95, p < 0.0001) (Figure 9D), with increasing *m* from *pre-ictal* to *ictal-onset* values directly causing a loss of neutral dynamics and the emergence of chaos (Figure 9C). Therefore, the increased network connectivity driving generalised seizures, also causes the emergence of chaotic dynamics. This would impair the network’s ability to maintain maximal memory about inputs, thus giving rise to network dysfunction during seizures (Haldeman & Beggs, 2005). Next, we investigated the effect of such chaotic dynamics on the response properties of the network.

### Dense Connectivity Impairs the Optimal Network Response Properties of Critical Networks

Another key property at criticality is that certain network response properties are optimised. In particular, evidence from *in vitro* and *in silico* studies have demonstrated that i) the network-mediated separation (NMS) – a network’s ability to discriminate between inputs (Bertschinger & Natschläger, 2004), and ii) the dynamic range (*δ*) – a network’s ability to represent a wide range of inputs (Kinouchi & Copelli, 2006; Shew et al., 2009), should be optimised at the critical point. Therefore, if generalised seizures emerge as a loss of criticality, one would expect emergent brain dysfunction due to impaired network response properties. Here, we assessed NMS and *δ* using the network model as we can disentangle the networks’ responses to inputs from its ongoing activity, something which is not possible *in vivo*.

**Figure 10:**
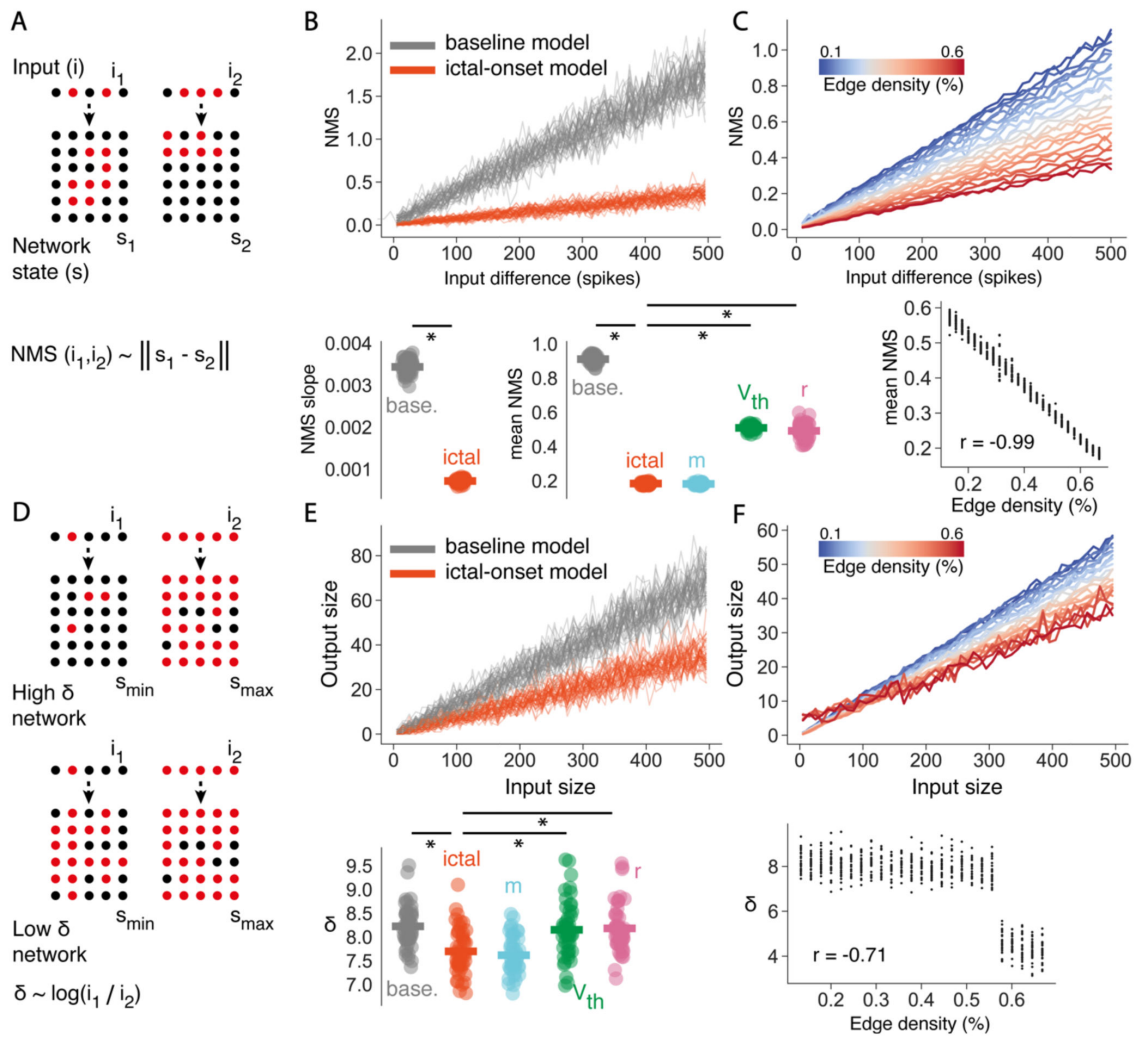
Increased connectivity disrupts the optimal network response properties of critical networks. (A) For 2 similar inputs (*i_1_* and *i_2_*) onto the network, the network mediated separation (NMS) is the Euclidean distance between the two corresponding network states (*s_1_* and *s_2_*). (B) NMS is shown as a function of input difference compared for *baseline* and *ictal-onset* full parameter models (top). NMS slope and mean NMS is compared for *baseline* and *ictal-onset* full models and single parameter models (*m = network connectivity, Vth = intrinsic excitability, r=synaptic strength*) (bottom). (C) NMS as a function of input difference for increasing network connectivity *m*, ranging from *pre-ictal* to *ictal-onset m* values (top). Correlation between *m* and mean NMS (bottom). (D) For different inputs, the dynamic range *δ* is the log ratio of the input sizes (*i_1_* and *i_2_*) that give rise to the largest and smallest network responses (*S_max_* and *S_min_*). (E) Output size as a function of input size compared for *baseline* and *ictal-onset* full parameter models (top). *δ* compared for *baseline* and *ictal-onset* full models and single parameter models (bottom). (F) Output size as a function of input size for increasing network connectivity *m*, ranging from *pre-ictal* to *ictal-onset m* values. Correlation between *m* and *δ* (bottom). * = p<0.01.

NMS is the property of a network to encode distinct inputs with distinct network states, enabling similar inputs to be separated and discriminated by a readout function (see Methods & Figure 10A). As predicted, *baseline* networks assume higher NMS values across the range of input sizes compared with *ictal-onset* networks (*baseline*: mean NMS = 0.91 ± 0.01, *ictal-onset*: 0.18 ± 0.00, U = 556991.0, p < 0.0001) (Figure 10B). This suggests an impaired ability of the network to discriminate between inputs during seizures. Furthermore, *ictal-onset* networks exhibit a significantly more shallow NMS slope (*baseline*: slope = 0.0034 ± 0.0000, *ictal-onset*: slope = 0.0007 ± 0.0000, t = 109.5, p < 0.0001) (Figure 10B). This indicates a reduced sensitivity of the NMS property to input size differences, demonstrating an impaired ability to distinguish between similar input pairs and highly different input pairs. Therefore, generalised seizures impair the ability of the brain to not only discriminate between inputs, but also represent input pairs according to their similarity.

Furthermore, to relate NMS reductions with underlying neuronal changes in seizures, we tested the link between NMS and *network connectivity*. We found that only the *network connectivity m* model produced NMS values matching the full model (0.18 ± 0.00, U = 3081443.4, p = 0.20), while *synaptic weights r* (0.49 ± 0.01, U = 1104945.0, p < 0.0001) and *intrinsic excitability V_th_* (0.51 ± 0.01, U = 1546742.0, p <0.0001) models failed to reduce NMS sufficiently to *ictal-onset* levels (Figure 10B). Furthermore, increasing the number of connections *m* resulted in a significantly correlated reduction in NMS statistics (mean NMS: r =- 0.99, p < 0.0001, NMS slope: r = -0.99, p < 0.0001), indicating a direct link between increased edge density and impaired NMS (Figure 10C).

We also investigated the dynamic range (*δ*), a measure of the range of input sizes that a system can represent (see Methods & Figure 10D). We found that *ictal-onset* networks exhibit significantly lower *δ* than *baseline* networks (*baseline*: 8.22 ± 0.06, *ictal-onset*: 7.70 ± 0.07, t = 5.83, p < 0.0001). This suggests that seizure networks exhibit a reduced capacity to represent a wide range of inputs, as expected in a network away from criticality. Once again, only the *network connectivity m* model matched the full *ictal-onset* model for *δ* (*m* : 7.62 ± 0.05, t = -0.95, p = 0.34; *r*. 8.18 ± 0.07, U = 593.0, p < 0.0001; *V_th_*: 8.15 ± 0.08, t = 4.28, p <0.0001) (Figure 10E). The parameter *network connectivity m* was also significantly negatively correlated with *δ* (r = -0.71, p < 0.0001), although we note a non-linear relationship (Figure 10F). This suggests that increased network connectivity during generalised seizures directly impairs the dynamics range.

Taken together, our model demonstrates that increased network connectivity impairs the optimal response properties of critical networks, resulting in reductions in NMS and dynamic range. Overall, this suggests that brain dysfunction emerging during generalised seizures is caused by a disruption of the optimal network response properties at criticality.

### Generalised Seizure Networks Are Inflexible Due to the Emergence of Sticky States

**Figure 11:**
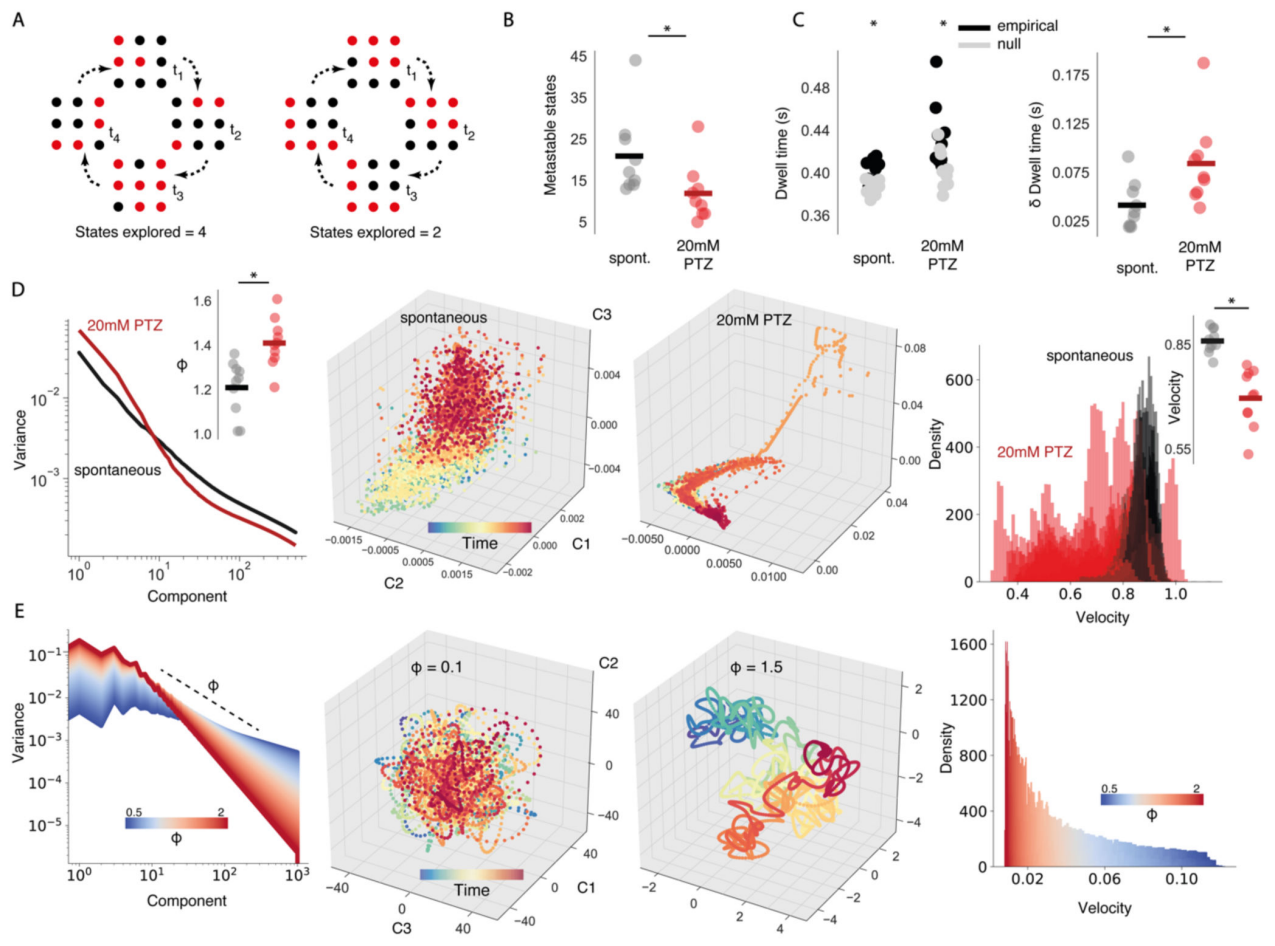
Generalised seizures cause sticky dynamics. (A) A critical system can explore a greater subset of its possible brain states (left), while a non-critical system will explore a more limited subset (right). (B) The number of metastable states compared across *spontaneous* (black bar = mean) and *20mM PTZ* conditions (red bar = mean). (C) Mean dwell time in each state compared across conditions, plotting both *empirical* and *null* model datapoints (left). Empirical dwell time minus null model dwell time (*δ* dwell time), as a measure of dwell time normalised to number of available states, compared across datasets (right). (D, left) Mean eigenspectrum function plotted across conditions, with Eigenspectrum slope *ϕ* plotted for each dataset (top right). (D, middle) 3d Isomap embedding of reconstructed attractor for an example fish. (D, right) State space velocity probability densities plotted for all fish, comparing *spontaneous* and *20mM PTZ* conditions, with mean velocity compared across datasets (top right). (E, left) Simulated eigenspectrum function plotted for increasing *ϕ*. (E, middle) Random projection of eigenspectra into state space for different *ϕ*. (E, right) State space velocity probability densities plotted as a function of *ϕ*. * = p<0.01

Finally, we investigated the impact of generalised seizures on state transition dynamics. A key property of systems at criticality is the ability to flexibly transition across a diversity of brain states – the number of states available to the system is maximal at the critical point (Haldeman & Beggs, 2005). Therefore, we theorised that generalised seizures should reduce the diversity of brain states, thus limiting the dynamic repertoire of the brain to a limit subset (Figure 11A). To this end, we compared the number of available states during *spontaneous* and generalised seizure (*20mM PTZ*) periods in empirical recordings. To do this we estimated the number of metastable states, defined as a set of state vectors that are more similar to one another than expected in a random system (see Methods). Interestingly, generalised seizure dynamics exhibit significantly fewer metastable states than spontaneous dynamics (*spontaneous:* 20.9 ± 3.30, *PTZ 20mM:* 11.9 ± 2.31, w = 1.9, p < 0.05) (Figure 11B). This indicates that generalised seizures limit the diversity of states the brain can enter into, which likely impairs the flexible transitioning across the full dynamic repertoire.

Next, we investigated the population mechanisms that might disrupt flexible dynamics. Having identified increased network connectivity as a driver of seizures, we reasoned that fewer brain states might be a natural consequence of the correlational structure of densely connected networks. We hypothesised that high multi-dimensional correlation in seizure networks would naturally limit the diversity of brain states, by restricting the space of possible neuronal activation patterns to a more limited subset. To test this, we use the eigenspectrum function which defines the amount of variance explained by the *n^th^* principal component, providing a measure of multi-dimensional correlation (see Methods). We found that the eigenspectrum slope *ϕ* significantly increased during generalised seizures (*spontaneous: ϕ* = 1.20 ± 0.04; *20mM PTZ*: *ϕ* = 1.41 ± 0.03, t = -6.05, p < 0.001), indicating greater variance captured in the first few components and increased multi-dimensional correlation (Figure 11D).

Interestingly, we note a direct relationship between *ϕ* and the velocity of the underlying dynamics. Here, we define velocity as the normalised Euclidean distance travelled per unit time across the whole neuronal population. As multi-dimensional correlation and therefore *ϕ* increases, population dynamics exhibit slower transitions in state space (Figure 11E). To visualise this, we simulate eigenspectra power laws and randomly project them into 3-dimensional space using a previously developed method (see Methods & Figure 11E) (Stringer et al., 2019). In line with this prediction, we find significantly slower dynamics during generalised seizures (in networks with high *ϕ*), compared with spontaneous activity (*spontaneous*: velocity = 0.86 ± 0.01, *20mM PTZ*: *velocity* = 0.70 ± 0.03, w = 0.0, p < 0.01) (Figure 11D). Slower dynamics occur due to earlier components dominating the variance, such that the variance of reconstructed trajectories in state space is driven by only a few key modes. Slower transitions in state space suggest the emergence of sticky states which are difficult to leave. This could explain reductions in state diversity, as sticky dynamics prevent the flexible transitioning into and out of the full dynamic repertoire.

For such sticky states to arise, we would expect longer times spent in each metastable state.

In line with this, we find significantly increased dwell times during generalised seizures (*spontaneous*: dwell time (s) = 0.40 ± 0.01, *20mM PTZ*: dwell time (s) = 0.43 ± 0.01, w = 1.0, p < 0.01) (Figure 11C). However, longer dwell times could occur by chance in systems with fewer states. Therefore, we calculated null models for each system, by evaluating the dwell time expected by chance. We find that the dynamics for spontaneous and generalised seizure periods are non-random, with longer dwell times than expected by chance (*spontaneous*: w= 0.0, p < 0.01, *20mM PTZ:* w = 0.0, p < 0.01) (Figure 11C). Importantly, generalised seizures show significantly longer dwell times even when accounting for the fewer available states (*spontaneous*: δ dwell time (s) = 0.02 ± 0.01, *20mM PTZ*: δ dwell time (s) = 0.03 ± 0.01, w = 2.0, p < 0.05) (Figure 11C).

Taken together, we find that the flexible dynamics of critical networks are impaired during generalised seizures, with a reduction in brain state diversity. Such inflexible dynamics may be caused by higher multi-dimensional correlation in dense networks, which gives rise to slow, sticky dynamics. Sticky, homogeneous dynamics would prevent flexible responses to inputs and impair the optimal exploration of semi-stable states required for scale-invariant dynamics at criticality, giving rise to brain dysfunction during seizures.

## Discussion

In this study, we estimated avalanche dynamics in whole-brain networks at neuronal resolution, to understand the effect of epileptic seizures on critical dynamics and emergent computation. Microscale avalanche dynamics showed that global network dynamics are driven away from a phase transition during seizures, a shift that was driven by increased density of network connections. Importantly, network connection density also regulated key computational properties that are optimised at criticality, giving rise to impaired network response properties and inflexible state transitions in seizure networks. Therefore, this study illustrates that criticality offers a unifying framework for understanding emergent brain dynamics and impairment during epileptic seizures.

### Understanding Epileptic Seizures Through the Lens of Criticality

Criticality describes a system in which dynamics are organised near to a phase transition, giving rise to favourable computational properties (Langton, 1990; Packard, 1988). A key property of critical systems are scale-invariant avalanches, propagating events that span the entire scale of the system (Sethna et al., 2001). Interestingly, scale-invariant avalanches have been reported in neuronal dynamics across multiple scales (Beggs & Plenz, 2003; Tagliazucchi et al., 2012), leading to the critical brain hypothesis. Studies of small neuronal populations first identified a link between seizures and criticality – drug-induced hyper-excitability gives rise to a loss of critical avalanche statistics (Beggs & Plenz, 2003; Bellay et al., 2015; Pasquale et al., 2008; Shew et al., 2009). These were later complemented by macroscale seizure recordings in epilepsy patients, which further suggested a deviation from the phase transition in coarse avalanche dynamics (Arviv et al., 2016; Meisel et al., 2012). However, bridging the gap between neuronal avalanches and macroscale dynamics, where seizures typically occur, has not previously been possible. We build on previous work by investigating avalanche dynamics across all spatial scales, from single neurons to entire brain regions. This is vital, because macroscale recordings mask the heterogeneity of single neuron activity (Keller et al., 2010; Meyer et al., 2018; Muldoon et al., 2013), thus obscuring neuronal avalanche dynamics. Conversely, inferring criticality in small neuronal populations is also challenging – subsampling larger networks can give rise to spurious critical statistics (Priesemann et al., 2009), while different brain regions can be differentially tuned to criticality (Suryadi et al., 2018). Given that we record from vastly greater numbers of neurons across all brain regions, avalanche dynamics will be more accurately assessed by mitigating subsampling and region-specific effects. The fact that whole-brain, neuronal-resolution critical statistics deviate dramatically during seizures suggests that microscale activity drives macroscale dynamics away from the phase transition.

However, we note that critical statistics are correlative measures and can appear in non-critical systems (Destexhe & Touboul, 2021; Touboul & Destexhe, 2017). Therefore, any inferences about the critical state of an observed system should be made with caution, as the network parameters defining the system’s dynamics are unknown and must be inferred indirectly.

The seizure avalanche dynamics observed in this study indicate the emergence of a supercritical regime, which predicts exponentially growing avalanches that saturate the macroscopic dynamics (Harris, 1963). This is corroborated by multi-scale seizure studies, that show propagating waves spreading throughout all microscale recording sites, before emerging at the macroscale (Martinet et al., 2017). However, these studies also suggest a more complicated picture than theory predicts – while some propagating events rapidly spread outwards from the ictal wavefront (Smith et al., 2016; Trevelyan et al., 2007), others are restrained by feedback inhibition, manifesting as postsynaptic potentials that fail activate their targets (Schevon et al., 2012; Trevelyan et al., 2006). This could explain the heterogeneous branching ratio values observed in this study, with transient departures from criticality emerging due to the push-and-pull between local excitation and inhibition.

Importantly, such a supercritical regime can explain emergent computational properties reported during epileptic seizures. In particular, we demonstrate that seizures exhibit an increase in chaotic dynamics, as predicted for a supercritical branching process (Haldeman & Beggs, 2005). Therefore, supercritical dynamics can explain the chaotic behaviour that has been reported in seizure networks, at the microscale using local field potentials (Hayashi & Ishizuka, 1995), and the macroscale via EEG recordings (Babloyantz & Destexhe, 1986). Such chaotic dynamics would reduce the network’s memory about recent inputs, due to high reverberating activity in the network.

Another key prediction from supercritical networks, is disruptions to flexible dynamics. Critical systems can engage in metastable dynamics manifesting as the transient formation and dissipation of diverse semi-stable states (Wildie & Shanahan, 2012), which supports flexible state transitions (Fingelkurts & Fingelkurts, 2001). We have shown that seizure dynamics exhibit reductions in brain state diversity, as predicted in supercritical networks. This can explain the reduced entropy of theta oscillatory rhythms recorded during hippocampal kindling in rats (Ren et al., 2021), and the emergence of highly stereotyped oscillatory dynamics from the seizure-onset zone in patients (Liu et al., 2018), which point to the emergence of abnormally attracting states that are frequently visited. Interestingly, reduced diversity of brain states is a key finding in medically-induced loss of consciousness studies in mice and human brain dynamics (Schartner et al., 2015; Wenzel, Han, et al., 2019). Therefore, a loss of metastable dynamics in supercritical networks, could explain the loss of consciousness that occurs during absence and tonic-clonic seizures (Blumenfeld, 2012).

Although we demonstrate a link between supercriticality and acute seizure impairments, further work is needed to show that chronic cognitive dysfunctions in epilepsy occur through similar mechanisms (Meador, 2002) – by studying avalanche dynamics in epilepsy models of chronic cognitive impairment (Shuman et al., 2020). One should also note that PTZ-induced GABA blockade does not capture the full diversity of seizure driving mechanisms in epilepsy (Rosch et al., 2019). Future work should therefore test the generalisability of supercriticality as a network regime for seizure dynamics using distinct genetic models of seizures (Baraban et al., 2013; Liao et al., 2019).

### Microscale Neuronal Network Mechanisms Driving Macroscale Seizure Dynamics

This study used whole-brain neuronal network modelling to infer the microscale alterations driving generalised seizure dynamics. Large scale models of epileptic networks have already demonstrated key microscale properties underlying seizures – such as inhibition exhaustion (Liou et al., 2020), excessive inhibitory to inhibitory coupling (Depannemaecker et al., 2022) and hub neurons (Hadjiabadi et al., 2021; Morgan & Soltesz, 2008). Here, we studied 3 key parameters – network connectivity, synaptic weights and intrinsic excitability, drawing inspiration from theoretical models of criticality (Zeraati et al., 2021), to relate predictions from criticality theory with observed seizure avalanche dynamics. Interestingly, modelling studies have shown that changing neuronal connections (Bornholdt & Roehl, 2003; Bornholdt & Rohlf, 2000; Tetzlaff et al., 2010; van Ooyen & Butz-Ostendorf, 2019), synaptic strengths (de Arcangelis et al., 2006; Rubinov et al., 2011) and intrinsic neuronal excitability (Levina et al., 2007), can stabilise brain dynamics to criticality. Importantly, these changes have all been implicated in the emergence of seizures, with changes in the number of axonal connections (Buckmaster, 2014; Buckmaster et al., 2002; Chu et al., 2010), the strengths of synapses (Geinisman et al., 1988; Poulter et al., 1999; Zhang et al., 2012) and the intrinsic excitability of neurons (Tokudome et al., 2016; Upreti et al., 2012; Vannini et al., 2020) linked to animal models of epilepsy.

We found that generalised seizure avalanches can be explained by increased neuronal connectivity. This is supported by large scale models of the rate dentate gyrus, which show that increased connections between granule cells can drive network hyper-excitability (Dyhrfjeld-Johnsen et al., 2007). However, we note that in our data, the formation of structural synapses is likely limited during the short timescales between pre-ictal and ictal periods following PTZ administration (Le Bé & Markram, 2006; Ozcan, 2017). Therefore, dense connectivity may instead be explained by the formation of new effective connections between neurons – these could arise through a diversity of mechanisms, such as volume transmitted GABA waves diffusing extracellularly (Magloire et al., 2022), or glia-glia coupling via gap junctions which can synchronise non-coupled neurons (Diaz Verdugo et al., 2019). However, a diversity of other microscale parameters not explored here have been shown to drive epileptic seizures – including intracellular Cl- (Huberfeld et al., 2007; Magloire et al., 2019; Shen et al., 2021; Staley, 2006), extracellular K+ (Florence et al., 2009; Uva et al., 2017; Ying et al., 2015), pro-ictal inhibition (Chang et al., 2018; Miri et al., 2018), distinct neuronal cell type dynamics (Khoshkhoo et al., 2017; Makinson et al., 2017), and cell death (Dingledine et al., 2014; Sloviter, 1987). Future work should use more specific parameterisations, to disentangle the differential role of each of these microscale parameters in driving excessive network connectivity during seizures.

In conclusion, we covered all spatial scales to investigate the collective behaviour of neuronal activity during seizures. In doing so we found evidence that networks deviate from criticality during seizures, and emerge as a chaotic state which impairs brain function. This investigation links the microscale population activity of neuronal networks with macroscale pathological brain dynamics that are characteristic of epileptic seizures.

## Supplementary Material

Movie 1 Spontaneous activity GCaMP6s calcium imaging of a single plane from a representative period of spontaneous activity. Acquisition speed = 2.73 frames/sec. Movie is played at 2× acquisition.

Movie 2 Brain activity following exposure to 5mM PTZ. GCaMP6s calcium imaging of a single plane from a representative period of brain activity following administration of 5mM PTZ. Acquisition speed = 2.73 frames/sec. Movie is played at 2× acquisition.

Movie 3 Brain activity following exposure to 20mM PTZ. GCaMP6s calcium imaging of a single plane from a representative period of brain activity following administration of 20mM PTZ. Acquisition speed = 2.73 frames/sec. Movie is played at 2× acquisition.

## Figures and Tables

**Table 1:** Key Resources Table

Regent or Resource	Source	Identifier
Tg(elavl3:H2B-GCaMP6s) (zebrafish line)	Freeman et al., 2014	ZDB-TGCONSTRCT-141023-1
Scanimage Software - Vidrio Technologies (acquisition)	This paper	https://docs.scanimage.org/
Suite2p (image registration and segmentation)	Pachitariu et al., 2017	https://github.com/MouseLand/suite2p
hidden markov model (calcium transient estimation)	Diana et al., 2019	https://github.com/giovannidiana
Brian2(Python network modelling software)	Stimberg et al., 2019	https://github.com/brian-team/brian2

## Significance Statement

Epileptic seizures are debilitating and impair normal brain function. It is unclear how the coordinated behaviour of neurons collectively impairs brain function during seizures. To investigate this we perform fluorescence microscopy in larval zebrafish, which allows for the recording of whole-brain activity at single-neuron resolution. Using techniques from physics, we show that neuronal activity during seizures drives the brain away from criticality, a regime that enables both high and low activity states, into an inflexible regime that drives high activity states. Importantly, this change is caused by more connections in the network, which we show disrupts the ability of the brain to respond appropriately to its environment. Therefore, we identify key neuronal network mechanisms driving seizures and concurrent cognitive dysfunction.
